# Association of air pollution with chronic obstructive pulmonary disease: anemia-related outcomes and exploratory genetic susceptibility analyses

**DOI:** 10.3389/fmed.2026.1925502

**Published:** 2026-09-08

**Authors:** Chi-Thien Dinh, Yueh-Lun Lee, Xuan Ngoc Tran, Kang-Yun Lee, Kian Fan Chung, Jer-Hwa Chang, Hsiao-Chi Chuang

**Affiliations:** 1 International Ph.D. Program in Medicine, College of Medicine, Taipei Medical University, Taipei, Taiwan; 2 Department of Internal Medicine, Faculty of Medicine, Can Tho University of Medicine and Pharmacy, Can Tho, Vietnam; 3 Department of Microbiology and Immunology, College of Medicine, Taipei Medical University, Taipei, Taiwan; 4 Division of Pulmonary Medicine, Department of Internal Medicine, Shuang Ho Hospital, Taipei Medical University, New Taipei City, Taiwan; 5 Graduate Institute of Clinical Medicine, College of Medicine, Taipei Medical University, Taipei, Taiwan; 6 Division of Pulmonary Medicine, Department of Internal Medicine, School of Medicine, College of Medicine, Taipei Medical University, Taipei, Taiwan; 7 National Heart and Lung Institute, Imperial College London, London, United Kingdom; 8 Royal Brompton & Harefield Hospitals, Guy’s and St Thomas’ NHS Foundation Trust, London, United Kingdom; 9 School of Respiratory Therapy, College of Medicine, Taipei Medical University, Taipei, Taiwan; 10 Division of Pulmonary Medicine, Department of Internal Medicine, Wan Fang Hospital, Taipei Medical University, Taipei, Taiwan; 11 Cell Physiology and Molecular Image Research Center, Wan Fang Hospital, Taipei Medical University, Taipei, Taiwan

**Keywords:** airflow limitation, anemia, gene-environment interaction (G × E), particulate matter, polygenic risk score (PRS), sulfur dioxide, Taiwan biobank (TWB)

## Abstract

**Background:**

Air pollution contributes to chronic obstructive pulmonary disease (COPD), but its associations with anemia-related outcomes in COPD and the modifying role of genetic susceptibility remain unclear.

**Objectives:**

To examine the associations of ambient air pollution with COPD and anemia-related outcomes, and to assess the potential modifying role of genetic susceptibility.

**Methods:**

This Taiwan Biobank case-control study included 7,566 cases and 22,698 age- and sex-matched controls. COPD was defined by presence of airflow obstruction or self-reported emphysema/chronic bronchitis, and anemia by World Health Organization hemoglobin cutoffs. Residential 1-, 3-, 6-, and 12-month exposures to particulate matter with an aerodynamic diameter ≤10 µm (PM_10_) and ≤2.5 µm (PM_2.5_), nitrogen dioxide (NO_2_), nitric oxide (NO), nitrogen oxides (NOx), sulfur dioxide (SO_2_), carbon monoxide (CO), and ozone (O_3_) were estimated at recruitment. Logistic regression was used for binary outcomes, and linear regression for spirometric and hematologic traits. Models were adjusted for age, sex, body mass index, smoking experience, and genetic principal components in genetic analyses.

**Results:**

Higher PM_10_, PM_2.5_, NO_2_, NO, NOx, SO_2_, and CO exposures were associated with higher COPD risk. Each 10 µg/m^3^ increase in PM_2.5_ was associated with COPD at 1 month [odds ratio (OR) = 1.252, 95% confidence interval (CI): 1.222–1.283] and 12 months (OR = 1.642, 95% CI: 1.588–1.699). Positive associations were observed when participants with COPD and anemia were compared with controls with anemia, whereas the within-COPD anemia analysis showed no consistent positive association between ambient air pollution and anemia status. Hemoglobin polygenic risk score showed nominal interactions with SO_2_ for anemia risk in COPD across all exposure periods (interaction ORs = 1.062–1.093). In the COPD genome-wide association study, rs4808295-A in *ZNF100* was associated with lower COPD risk (OR = 0.865, *p* = 1.63 × 10^−10^) and remained stable in sensitivity analysis.

**Conclusions:**

Residential air pollution exposure across the evaluated pre-recruitment averaging periods was associated with COPD. The nominal HGB PRS-SO_2_ interaction suggests heterogeneity in hematologic responses according to genetic susceptibility, whereas the within-COPD anemia analysis did not show a consistent positive association between ambient air pollution and anemia status.

## Graphical Abstract

**Figure:**
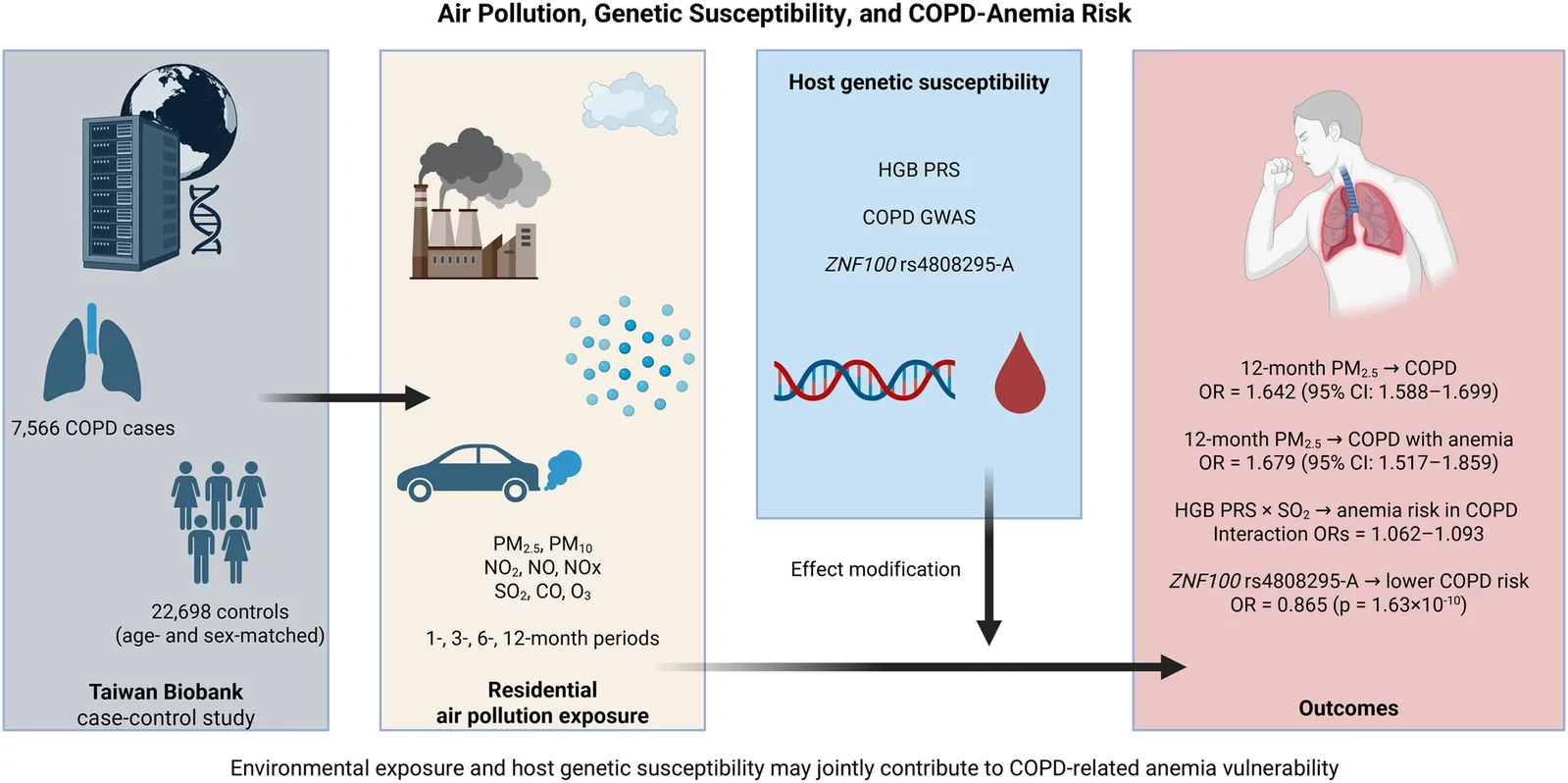

## 1 Introduction

Chronic obstructive pulmonary disease (COPD) is a major global health burden, affecting an estimated 10.3% of adults aged 30–79 years worldwide [95% confidence interval (CI): 8.2%–12.8%] (1) and causing 3.5 million deaths in 2021 (2). Although cigarette smoking remains the leading risk factor, ambient air pollution is increasingly recognized as an important environmental contributor to COPD development and progression. Other factors, including age, sex, BMI, occupational and socioeconomic conditions, comorbidities, and nutritional status, may also influence COPD and anemia risk and complicate the observed associations with air pollution (3–5). A systematic review and meta-analysis found that each 10 μg/m^3^ increase in long-term exposure to particulate matter with an aerodynamic diameter ≤ 2.5 µm (PM_2.5_) was associated with an 18% higher risk of incident COPD, while long-term nitrogen dioxide (NO_2_) exposure was associated with a 7% higher risk (6). Consistent evidence from Taiwan showed that reductions in PM_2.5_ exposure were associated with lower COPD risk and slower lung function decline (7). However, not all individuals exposed to similar levels of air pollution develop COPD, suggesting that susceptibility to pollution-related respiratory injury may differ across individuals.

COPD is increasingly recognized as a systemic disease rather than an isolated airway disorder, and anemia is a common comorbidity in this setting (3, 4). Reported anemia prevalence in COPD ranges from 7.5% to 34%, and anemia has been associated with worse symptom burden, reduced exercise capacity, more frequent exacerbations, higher readmission risk, and increased mortality (5, 8–10). Air pollution has also been associated with anemia-related phenotypes. Long-term exposure to particulate matter with an aerodynamic diameter ≤ 10 µm (PM_10_) has been associated with lower hemoglobin (HGB) levels (11), while a recent meta-analysis showed each 10 μg/m^3^ increase in PM_2.5_ and NO_2_ was associated with 20.0% and 12.7% higher anemia risk, respectively (12). However, whether these hematologic effects of air pollution differ in the context of COPD remains unclear, supporting the need to evaluate anemia-related outcomes specifically among individuals with COPD (3, 8). Air pollution may contribute to anemia in COPD through overlapping inflammatory and oxidative pathways. Inhaled pollutants can aggravate pulmonary and systemic inflammation, while COPD itself is characterized by systemic inflammatory effects that may impair iron utilization and erythropoietic responses. Oxidative stress may also increase erythrocyte injury and alter red blood cell survival. Together, these processes provide a biologically plausible basis for evaluating anemia-related outcomes among individuals with COPD exposed to ambient air pollution (3, 8, 11, 12).

Individuals exposed to similar levels of air pollution do not necessarily develop COPD or anemia-related manifestations to the same extent, suggesting differences in host susceptibility. Genetic variation may contribute to this heterogeneity by influencing lung function, COPD susceptibility, hemoglobin levels, and other hematologic traits. Polygenic risk scores summarize the cumulative contribution of multiple susceptibility variants, whereas GWAS can identify individual loci associated with disease or hematologic phenotypes. Gene-environment interaction analyses further assess whether associations with air pollution differ according to genetic background (13–16). Despite growing evidence that air pollution is associated with COPD and anemia-related outcomes separately, few studies have examined these conditions within a common analytical framework or evaluated whether host genetic susceptibility modifies their associations with environmental exposure. Evidence is particularly limited for individuals with co-occurring COPD and anemia and for East Asian populations, in whom genetic architecture and PRS performance may differ from predominantly European cohorts (6, 12, 17). The novelty of this study lies in integrating residential air pollution exposure across several pre-recruitment averaging periods with COPD and anemia-related outcomes, externally derived PRSs, GWAS, and gene-environment interaction analyses in the Taiwan Biobank. Accordingly, the objective of this study was to investigate the associations of air pollution exposure with COPD and anemia-related outcomes, with a particular focus on COPD accompanied by anemia, and to determine whether genetic susceptibility modifies these associations.

## 2 Methods

### 2.1 Ethical considerations and data source

This study was approved by the Taipei Medical University Joint Institutional Review Board (Taipei, Taiwan; TMU-JIRB No. N202003075). Analyses used de-identified Taiwan Biobank (TWB) data, with all participants having provided written informed consent at enrollment (18). Additional informed consent for this analysis was waived because only de-identified data were used.

### 2.2 Study design and population

**Figure 1:**
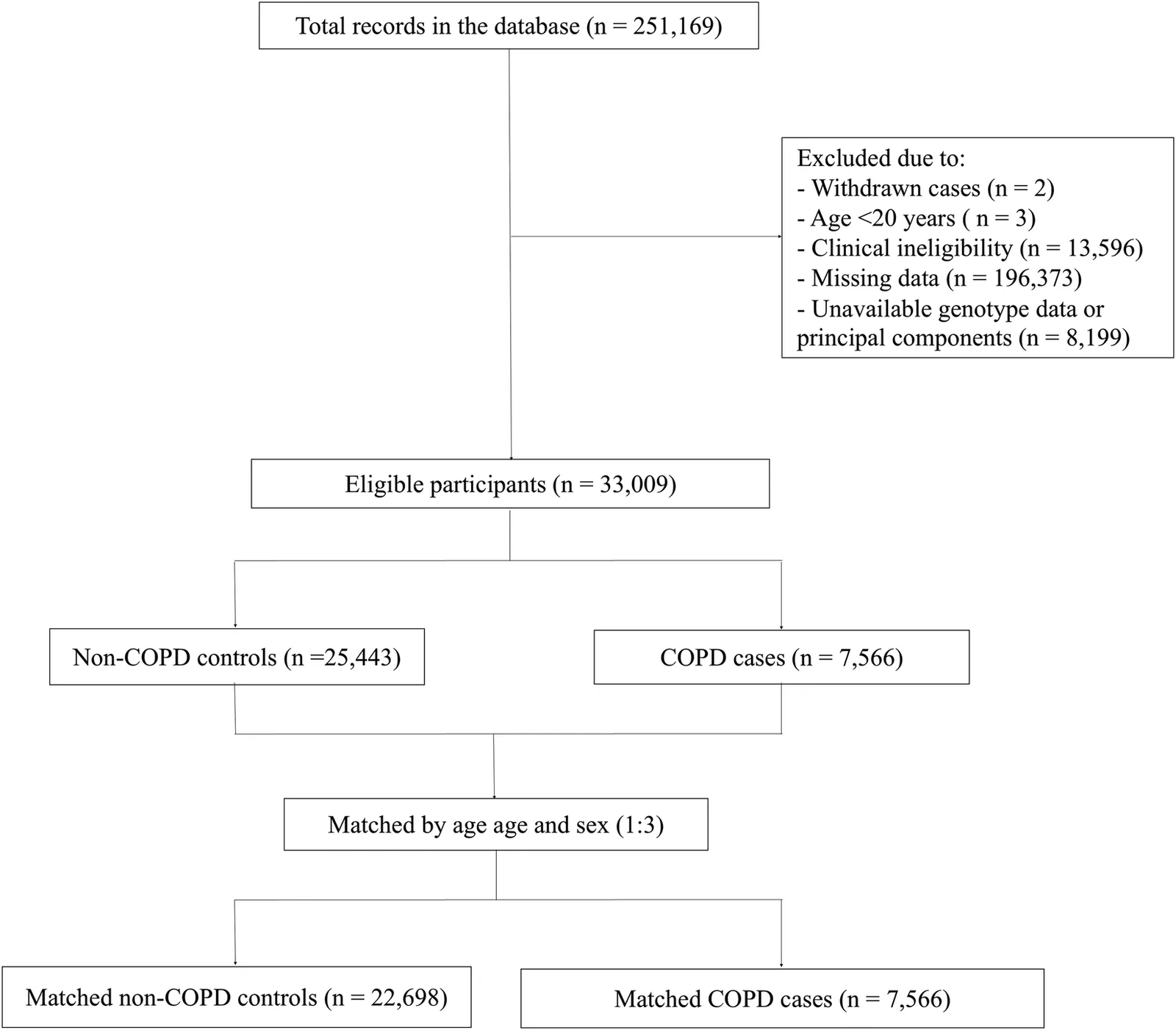
Flowchart of study design.

This frequency-matched case-control study used genotype and phenotype data from participants recruited between 2012 and 2019 from the TWB (https://www.twbiobank.org.tw/). Comprehensive descriptions of the TWB design, data acquisition methods, genotyping approaches, and quality control procedures are available in previously published studies (19). COPD was defined as either spirometry-defined airflow obstruction, based on a ratio of forced expiratory volume in 1 s to forced vital capacity (FEV_1_/FVC ratio) < 0.70 (4), or self-reported emphysema or chronic bronchitis. Participant-level information on bronchodilator administration and manoeuvre-level spirometry quality criteria was not available in the analytic dataset. The combined definition incorporated complementary spirometric and questionnaire-based phenotype information and was applied consistently before matching and across all subsequent analyses. Participants who met neither criterion were classified as controls. Individuals with cancer, renal failure, asthma, or missing data required for phenotype definition, exposure assessment, or covariate adjustment were excluded before matching. After removing individuals with ineligible data, the eligible population consisted of 7,566 cases and 25,443 controls, corresponding to an approximate case-control ratio of around 1:3. Cases and controls were matched on age and sex using coarsened exact matching (CEM), followed by 1:3 control sampling. The details of participant selection, flowchart of study design, as well as CEM procedure are provided in Figure 1 and S1, S2 of Supplementary Information.

### 2.3 Air pollution exposure assessment

Air pollution data were obtained from the Taiwan Air Quality Monitoring Network operated by the Ministry of Environment, Taiwan. During the recruitment period, the national network comprised approximately 76–77 fixed-site monitoring stations, with the number of stations contributing data varying slightly by pollutant and calendar year. Concentrations of PM_10_, PM_2.5_, NO_2_, nitric oxide (NO), nitrogen oxides (NOx), sulfur dioxide (SO_2_), carbon monoxide (CO), and ozone (O_3_) were obtained from the monitoring database (20). Air quality monitoring data were subject to the quality assurance and quality control procedures of the Taiwan Air Quality Monitoring Network. According to the Ministry of Environment monitoring criteria, an hourly value was considered valid when at least 45 min of valid sampling were available, a daily mean required at least 16 valid hourly values, and a monthly mean required at least 20 valid daily values (20). We additionally assessed the availability of monthly monitoring data by pollutant, monitoring station, and calendar year during 2012–2019. For each pollutant-station-year combination, monthly completeness was defined as the number of months with an available concentration value divided by 12. Completeness was subsequently summarized by pollutant and calendar year and is reported in Supplementary Table S1. Individual residential exposure was assigned according to each participant's residential location and recruitment date. Residential locations were geocoded, and the nearest fixed-site air quality monitoring station was identified for each residential location. Pollutant measurements from the corresponding monitoring station were then matched to each participant according to the recruitment date, consistent with the station-based exposure-assignment approach used in previous Taiwan Biobank studies (21, 22). For each participant, monthly pollutant concentrations preceding recruitment were used to calculate moving-average exposures over 1, 3, 6, and 12 months. These exposure windows were selected to evaluate associations across different pre-recruitment averaging periods. The 1-month average represented relatively recent exposure, which may capture shorter-lag respiratory and systemic responses such as inflammation and oxidative stress, whereas the 3- and 6-month averages represented intermediate averaging periods and the 12-month average provided a broader summary of repeated residential ambient exposure before recruitment (23–25). Similar 1-, 3-, 6-, and 12-month exposure intervals have previously been applied in Taiwan to evaluate respiratory-related outcomes using a monitoring-station-based exposure approach (26). Because all four moving averages ended at the recruitment date and overlapped by construction, they were analyzed separately and were not interpreted as independent temporal effects, cumulative lifetime exposure, or specific etiologic latency periods. Correlations among the four exposure windows were therefore quantified separately for each pollutant.

### 2.4 Polygenic risk score analyses

PRS were derived from external resources rather than from the internal GWAS results of the present study (27). For COPD risk analyses, external PRSs for FEV_1_/FVC (PGS000210) (28) and COPD (PGS001332) (29), obtained from the Polygenic Score Catalog (https://www.pgscatalog.org/), were calculated in the TWB target genotype dataset using PLINK v2.0 and standardized as z-scores before analysis. For anemia-related analyses, hematologic PRSs were constructed using external East Asian summary statistics derived from BioBank Japan/PheWeb (https://pheweb.jp/) (30). Trait-specific PRS were generated using PRSice v2.3.5 under the clumping-and-thresholding (C + T) framework (17, 27). Single-nucleotide polymorphisms (SNPs) were first selected according to GWAS association *p*-values and then linkage disequilibrium-pruned using a 250-kb clumping period and an r^2^ threshold of 0.1 (31). For each hematologic trait, the primary PRS was calculated as the full clumped score including variants with GWAS *P* ≤ 1, thereby avoiding *p*-value threshold optimization within the target dataset (27). Scores were calculated in the TWB genotype dataset, merged into the matched analytic sample, and standardized as z-scores. Because this study focused on gene-environment interaction effects rather than disease risk prediction, PRSs were interpreted as standardized genetic predisposition indices. Details of PRS construction and analyses are provided in S3 of Supplementary Information.

### 2.5 Genome-wide association analyses and single-variant gene-air pollution interaction analyses

GWAS analysis was performed in PLINK v1.9 under an additive logistic regression model adjusted for age, sex, body mass index (BMI), smoking experience, and principal components 1–10 (PC1-PC10). The primary GWAS analysis evaluated COPD risk in the matched case-control sample and anemia risk within the COPD subgroup. A sensitivity GWAS additionally applied variant-level filters for minor allele frequency, genotype missingness, and Hardy-Weinberg equilibrium. Genome-wide significance was defined as *P* < 5 × 10^−8^; suggestive and exploratory thresholds were defined as *P* < 1 × 10^−6^ and *P* < 1 × 10^−5^, respectively (32). Independent loci were identified by linkage disequilibrium clumping. Manhattan plots, quantile-quantile plots, and genomic inflation factors were used to evaluate GWAS results, and lead loci were annotated using dbSNP, Ensembl, and the GWAS Catalog. Single-variant interaction analyses were conducted using lead SNPs identified from the COPD and anemia-in-COPD GWAS. Additive genetic coding was used primarily, whereas a dominant model was applied when homozygous minor-allele carriers were sparse. Logistic regression models included air pollution exposure, the lead SNP, their interaction term, and covariates. The detailed GWAS workflow is provided in S4 of Supplementary Information.

### 2.6 Statistical analysis

Continuous variables were summarized as means and standard deviations (SDs) and categorical variables as frequencies and percentages. Extreme continuous values were winsorized at |*Z*| > 4 before model fitting. Group comparisons used Student's *t*-test for continuous variables and chi-square test or Fisher's exact test for categorical variables, as appropriate. For binary outcomes, logistic regression was used to estimate ORs and 95% confidence intervals (CIs). Primary environmental association models examined air pollution exposure and COPD risk with adjustment for age, sex, BMI, and smoking experience (ever vs. never). Smoking experience was included *a priori* because tobacco use is a major determinant of COPD. This harmonized cohort-wide variable was available for the full matched analytic sample and was used consistently across the primary environmental and genetic models. To further evaluate potential residual confounding by tobacco exposure, a sensitivity analysis was conducted among participants with complete detailed smoking information. In this analysis, smoking was classified as never, former, or current smoking, and the models additionally adjusted for smoking duration and smoking intensity. Smoking duration was expressed in years, and smoking intensity was standardized to packs per day according to the reported daily, weekly, or monthly smoking frequency. Each pollutant and exposure-averaging period was evaluated in a separate regression model. This prespecified single-pollutant framework provided a consistent basis for comparing associations across pollutants and avoided coefficient instability arising from the simultaneous inclusion of correlated pollutants with shared emission sources. Accordingly, the estimates represent associations with individual pollutants as indicators of ambient pollution exposure rather than mutually independent pollutant effects. Genetic analyses, including PRS-based and single-variant interaction models, additionally adjusted for PC1-PC10 to account for population structure. Gene-environment interactions were evaluated by adding cross-product terms between each environmental factor and the PRS or SNP to the corresponding regression models. Linear regression was used for continuous outcomes, including FEV_1_/FVC, HGB, red blood cell (RBC), hematocrit, mean corpuscular volume (MCV), mean corpuscular hemoglobin (MCH), and mean corpuscular hemoglobin concentration (MCHC), to estimate *β* coefficients and 95% CIs. Anemia was defined by World Health Organization criteria as HGB concentration <13 g/dL in men and <12 g/dL in women (33). Anemia was analyzed as a binary hematologic phenotype; etiologic subtyping was not performed because iron-status, inflammatory, and vitamin-related biomarkers were not available in the analytic dataset. For the overall anemia analysis, anemia was modeled as the binary outcome in the full matched analytic sample regardless of COPD status, with participants without anemia serving as the reference group. A separate within-COPD anemia analysis compared participants with COPD and anemia with participants with COPD without anemia. Pearson correlation coefficients were calculated among the 1-, 3-, 6-, and 12-month exposure averages separately for each pollutant to quantify the degree of overlap among the prespecified exposure summaries. Inter-pollutant correlations were also evaluated using Pearson correlation coefficients separately within each pre-recruitment exposure-averaging period. The overlapping exposure averages were evaluated in separate regression models and were not interpreted as independent temporal effects. PM_10_ and PM_2.5_ were scaled per 10-unit increase, whereas other pollutants were analyzed in their original units. A complete-case approach was applied. Participants without the exposure estimates, phenotype information, or covariate data required for the analyses were excluded before matching, and missing air pollution values were not imputed. Analyses were performed using Python 3. Outside the GWAS analyses, two-sided *P* values <0.05 were treated as nominal evidence; no formal multiplicity correction was applied, and interaction findings were interpreted as exploratory. To assess the robustness of the findings to COPD ascertainment, a sensitivity analysis was restricted to participants with spirometry-defined airflow obstruction, with participants meeting neither the spirometric nor self-reported criterion retained as controls.

## 3 Results

### 3.1 Baseline characteristics and exposure profile

Table 1 presents the baseline characteristics of the matched study population. Age and sex were comparable between the matched groups. The prevalence of never smoking was also nearly identical between controls and COPD cases (72.3% vs. 72.2%, *p* = 0.935), indicating strong baseline comparability in smoking experience. BMI was lower in the COPD group than in controls (23.93 ± 3.60 vs. 24.23 ± 3.76 kg/m^2^). Among the 7,566 participants classified as having COPD, 7,269 (96.1%) met the spirometry-defined airflow-obstruction criterion. Of these, 7,169 (94.8% of all COPD cases) met the spirometric criterion only and 100 (1.3%) met both the spirometric and self-reported criteria; only 297 participants (3.9%) were classified as COPD solely on the basis of self-report (Supplementary Table S2). As expected, pulmonary function was lower in the COPD group, including FEV_1_% predicted (55.44 ± 20.06 vs. 93.55 ± 16.03) and FEV_1_/FVC ratio (51.94 ± 14.99% vs. 86.13 ± 6.05%). Among participants with spirometry-defined airflow obstruction, 50.2% had FEV_1_ values of 50%–79% predicted, 32.7% had values of 30%–49% predicted, 10.1% had values <30% predicted, and 7.1% had values ≥80% predicted. These FEV_1_-based categories are presented descriptively and should not be interpreted as formal GOLD severity stages. The COPD group showed lower RBC count, HGB, and MCHC, but higher hematocrit and MCV than controls. Table 2 shows that the COPD group had higher exposure to most measured pollutants across exposure periods. PM_2.5_ exposure was higher in COPD cases than controls at each exposure period, with the largest between-group difference at 12 months (25.25 ± 7.63 vs. 22.23 ± 7.65 µg/m^3^). Higher exposure in COPD cases than controls was also observed for PM_10_, NO_2_, NO, NOx, SO_2_, and CO, whereas O_3_ was lower at 1-, 3-, and 6-month periods but not 12 months. Overall, the COPD group had poorer lung function, differences in erythrocyte indices, and generally higher pollutant exposure. Within-pollutant correlations across the four pre-recruitment exposure averages ranged from 0.454 to 0.978 (Supplementary Table S3). Correlations were generally stronger between temporally closer averaging periods, whereas greater differences were observed between the 1- and 12-month averages for PM_10_ (*r* = 0.657), PM_2.5_ (*r* = 0.592), and O_3_ (*r* = 0.454). Inter-pollutant correlations also indicated substantial shared variation among several pollutants (Supplementary Table S4). The strongest correlations were observed for NO_2_ and NOx (*r* = 0.934–0.944), PM_10_ and PM_2.5_ (*r* = 0.883–0.896), and NOx and CO (*r* = 0.887–0.919) across the four averaging periods. SO_2_ showed moderate positive correlations with several particulate and combustion-related pollutants, including PM_2.5_ (*r* = 0.450–0.595), NO_2_ (*r* = 0.549–0.652), and NOx (*r* = 0.484–0.533).

**Table 1:** Baseline characteristics of study subjects (mean ± SD), matched by age and sex.

Characteristics	Control (*n* = 22,698)	COPD (*n* = 7,566)	*p*-value
Age, year	50.90 ± 10.86	50.85 ± 10.94	0.732
Male, *n* (%)	8,058 (35.5%)	2,686 (35.5%)	1.000
BMI, kg/m^2^	24.23 ± 3.76	23.93 ± 3.60	**<0** **.** **05**
Never smoking, *n* (%)	16,402 (72.3%)	5,463 (72.2%)	0.935
Pulmonary function test			
FVC, L	2.83 ± 0.79	2.76 ± 0.84	**<0** **.** **05**
FVC, % predicted	93.23 ± 15.77	91.17 ± 18.91	**<0** **.** **05**
FEV_1_, L	2.43 ± 0.70	1.43 ± 0.61	**<0** **.** **05**
FEV_1_, % predicted	93.55 ± 16.03	55.44 ± 20.06	**<0** **.** **05**
FEV_1_/FVC ratio, %	86.13 ± 6.05	51.94 ± 14.99	**<0** **.** **05**
FEV_1_-based spirometric severity categories		(*n* = 7,269)	
≥80%	–	513 (7.06)	–
50%–79%	–	3,646 (50.16)	–
30%–49%	–	2,373 (32.65)	–
<30%	–	737 (10.14)	–
Erythrocyte indices			
RBC count, 10^6^/µL	4.74 ± 0.52	4.72 ± 0.51	**<0** **.** **05**
HGB, g/dL	13.74 ± 1.59	13.70 ± 1.59	**<0** **.** **05**
Anemia, *n* (%)	2,612 (11.5%)	896 (11.8%)	0.443
Hematocrit, %	41.47 ± 4.19	41.77 ± 4.32	**<0** **.** **05**
MCV, fL	87.85 ± 7.73	88.84 ± 8.14	**<0** **.** **05**
MCH, pg	29.12 ± 3.02	29.12 ± 2.93	0.949
MCHC, g/dL	33.12 ± 1.63	32.78 ± 1.67	**<0** **.** **05**

BMI, body mass index; FVC, forced vital capacity; FEV_1_, forced expiratory volume in 1 s; RBC, red blood cell; HGB, hemoglobin; MCV, mean corpuscular volume; MCH, mean corpuscular hemoglobin; MCHC, mean corpuscular hemoglobin concentration. Bold values indicate statistically significant results (*p* < 0.05).

**Table 2:** Air pollution exposure concentrations (mean ± SD).

Exposure variable	Control (*n* = 22,698)	COPD (*n* = 7,566)	*p*-value
PM_10_			
1-month, µg/m^3^	42.37 ± 19.17	47.89 ± 20.85	**<0**.**05**
3-month, µg/m^3^	43.18 ± 17.65	48.27 ± 18.94	**<0**.**05**
6-month, µg/m^3^	45.29 ± 16.54	50.27 ± 17.28	**<0**.**05**
12-month, µg/m^3^	46.10 ± 14.84	52.14 ± 15.21	**<0**.**05**
PM_2.5_			
1-month, µg/m^3^	20.10 ± 10.00	22.58 ± 11.01	**<0**.**05**
3-month, µg/m^3^	20.58 ± 9.28	22.74 ± 9.98	**<0**.**05**
6-month, µg/m^3^	21.77 ± 8.63	23.87 ± 8.94	**<0**.**05**
12-month, µg/m^3^	22.23 ± 7.65	25.25 ± 7.63	**<0**.**05**
NO_2_			
1-month, ppb	13.66 ± 6.10	15.18 ± 6.44	**<0**.**05**
3-month, ppb	13.89 ± 5.84	15.19 ± 6.04	**<0**.**05**
6-month, ppb	14.45 ± 5.59	15.61 ± 5.68	**<0**.**05**
12-month, ppb	14.56 ± 5.17	15.88 ± 5.22	**<0**.**05**
NO			
1-month, ppb	3.59 ± 5.39	4.56 ± 5.60	**<0**.**05**
3-month, ppb	3.61 ± 4.63	4.57 ± 5.16	**<0**.**05**
6-month, ppb	3.70 ± 4.52	4.67 ± 5.20	**<0**.**05**
12-month, ppb	3.74 ± 4.16	4.73 ± 4.81	**<0**.**05**
NOx			
1-month, ppb	17.22 ± 10.09	19.88 ± 11.32	**<0**.**05**
3-month, ppb	17.44 ± 9.49	19.79 ± 10.23	**<0**.**05**
6-month, ppb	18.13 ± 9.14	20.31 ± 9.76	**<0**.**05**
12-month, ppb	18.43 ± 8.69	20.67 ± 9.31	**<0**.**05**
SO_2_			
1-month, ppb	2.94 ± 1.22	3.34 ± 1.29	**<0**.**05**
3-month, ppb	2.94 ± 1.17	3.36 ± 1.23	**<0**.**05**
6-month, ppb	2.97 ± 1.14	3.42 ± 1.20	**<0**.**05**
12-month, ppb	3.03 ± 1.12	3.49 ± 1.21	**<0**.**05**
CO			
1-month, ppm	0.41 ± 0.19	0.46 ± 0.21	**<0**.**05**
3-month, ppm	0.42 ± 0.17	0.46 ± 0.19	**<0**.**05**
6-month, ppm	0.44 ± 0.16	0.47 ± 0.18	**<0**.**05**
12-month, ppm	0.44 ± 0.15	0.48 ± 0.17	**<0**.**05**
O_3_			
1-month, ppb	28.73 ± 6.88	28.43 ± 7.23	**<0**.**05**
3-month, ppb	28.56 ± 5.18	28.27 ± 5.30	**<0**.**05**
6-month, ppb	28.71 ± 3.98	28.44 ± 3.87	**<0**.**05**
12-month, ppb	28.82 ± 3.32	28.87 ± 3.46	0.339

PM_10_, particulate matter with an aerodynamic diameter ≤ 10 µm; PM_2.5_, particulate matter with an aerodynamic diameter ≤ 2.5 µm; NO_2_, nitrogen dioxide; NO, nitric oxide (nitrogen monoxide); NOx, nitrogen oxides; SO_2_, sulfur dioxide; CO, carbon monoxide; O_3_, ozone. Bold values indicate statistically significant results (*p* < 0.05).

### 3.2 Associations between air pollution exposure and COPD- and anemia-related outcomes

**Figure 2:**
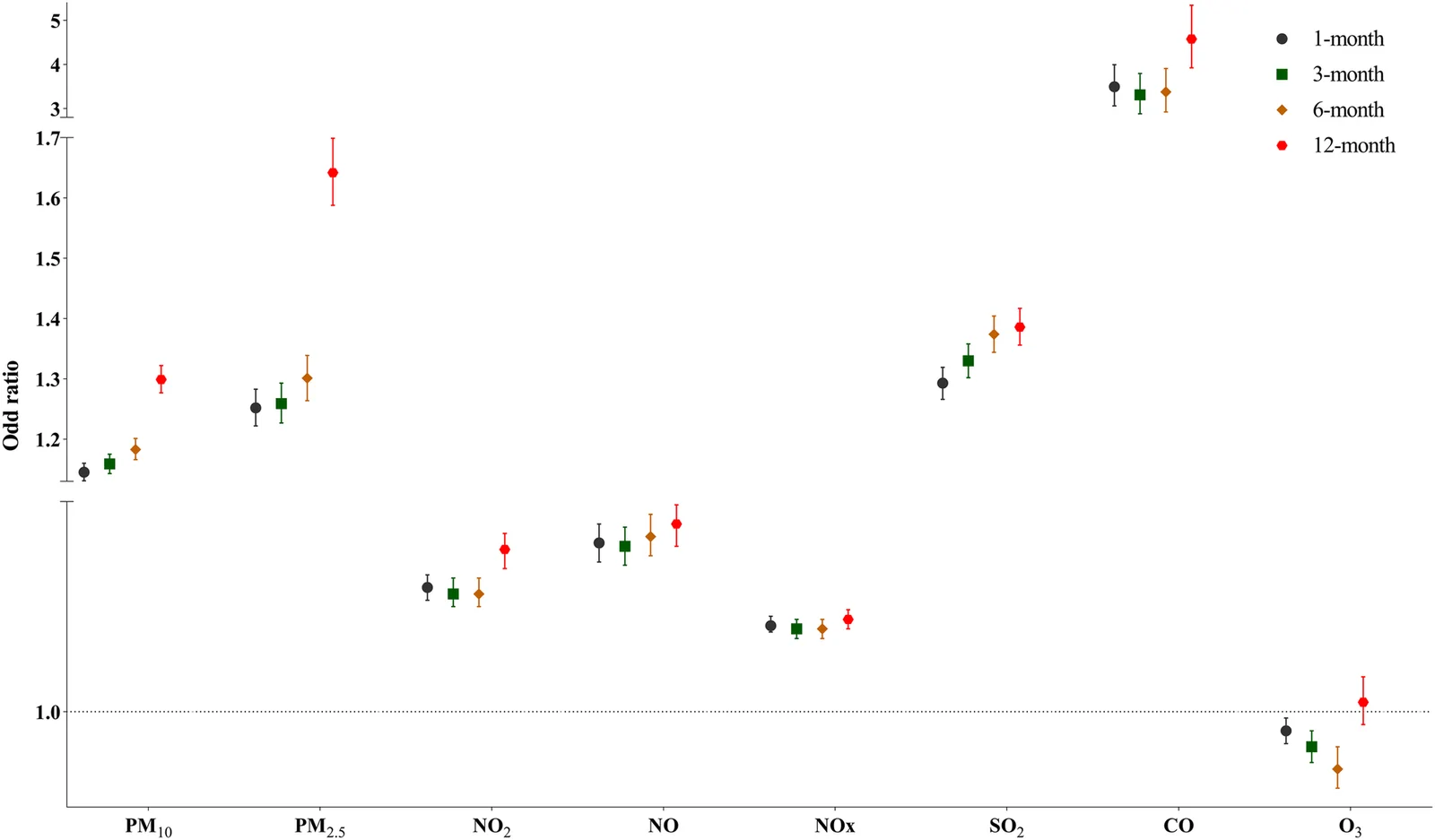
Associations between air pollution exposure and COPD risk. Odds ratios (ORs) indicate the risk of COPD based on logistic regression models, per 10 µg/m^3^ increase in PM_10_ and PM_2.5_ concentrations, and per 1-unit increase in concentrations of other pollutants (NO_2_, NO, NOx, SO_2_, CO, O_3_), adjusted for age, sex, BMI, and smoking experience. Black, green, orange, and red symbols indicate the 1-, 3-, 6-, and 12-month exposure periods, respectively, and error bars indicate 95% confidence intervals (CIs).

**Figure 3:**
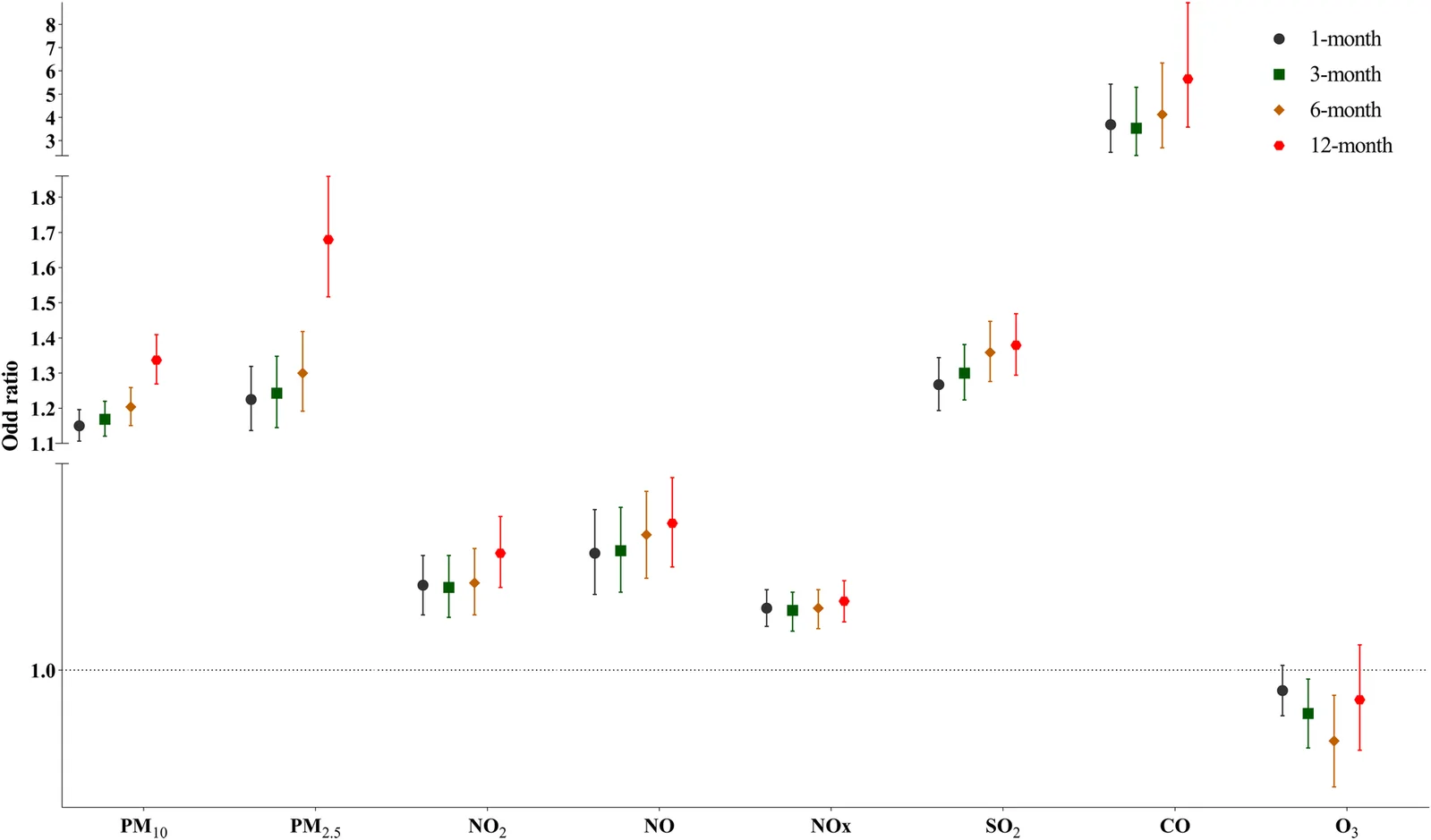
Associations between air pollution exposure and COPD status among participants with anemia (COPD with anemia vs. controls with anemia). Odds ratios (ORs) indicate the risk of COPD based on logistic regression models, per 10 µg/m^3^ increase in PM_10_ and PM_2.5_ concentrations, and per 1-unit increase in concentrations of other pollutants (NO_2_, NO, NOx, SO_2_, CO, O_3_), adjusted for age, sex, BMI, and smoking experience. Black, green, orange, and red symbols indicate the 1-, 3-, 6-, and 12-month exposure periods, respectively, and error bars indicate 95% confidence intervals (CIs).

Figure 2 and Supplementary Table S5 show higher exposure to most pollutants was associated with higher COPD risk. For several pollutants, the estimated ORs differed across averaging periods and were numerically larger for the 12-month than the 1-month average. For PM_2.5_, each 10 µg/m^3^ increase was associated with adjusted ORs of 1.252 (95% CI: 1.222–1.283) at 1 month and 1.642 (95% CI: 1.588–1.699) at 12 months. Positive associations were also observed for PM_10_, NO_2_, NO, NOx, SO_2_, and CO. In contrast, O_3_ showed inverse association at the 1-, 3-, and 6-month periods, whereas the 12-month association was not significant. In a sensitivity analysis restricted to participants with complete detailed smoking information (*n* = 27,696), additional adjustment for smoking duration and intensity produced materially unchanged associations between air pollution exposure and COPD (Supplementary Table S6). The environmental associations were maintained when the analysis was restricted to the 7,269 participants with spirometry-defined airflow obstruction (Supplementary Table S7). For PM_2.5_, the adjusted ORs were 1.262 (95% CI: 1.231–1.294) at 1 month and 1.671 (95% CI: 1.614–1.729) at 12 months, closely corresponding to the estimates from the primary combined COPD definition. Similar patterns were observed for the other pollutants. Associations according to the mutually exclusive COPD ascertainment subgroups are presented in Supplementary Table S8; estimates for the small self-report-only and combined-criteria subgroups were generally less precise than those for the spirometry-defined subgroup. Figure 3 and Supplementary Table S9 show positive associations when participants with COPD and anemia were compared with controls with anemia as the reference group. For PM_2.5_, each 10 µg/m^3^ increase was associated with the adjusted ORs of 1.225 (95% CI: 1.137–1.319) at 1 month and 1.679 (95% CI: 1.517–1.859) at 12 months. Positive associations were also observed for PM_10_, NO_2_, NO, NOx, SO_2_, and CO, whereas O_3_ showed inverse associations only at the 3 and 6 months. By contrast, in the overall anemia analysis, which included the full matched sample regardless of COPD status, with participants without anemia as the reference group, no consistent positive association with ambient air pollution was observed (Supplementary Table S10). For PM_2.5_, each 10 µg/m^3^ increase was associated with the adjusted ORs of 0.944 (95% CI: 0.911–0.979) at 1 month, 0.935 (95% CI: 0.900–0.972) at 3 months, and 0.952 (95% CI: 0.914–0.993) at 6 months. Most gaseous pollutants were not significantly associated with anemia risk across exposure periods, although O_3_ showed inverse associations at the 3- and 6-month periods. To directly evaluate anemia status within COPD, an additional analysis compared COPD participants with anemia (*n* = 896) with those without anemia (*n* = 6,670). No consistent positive associations were observed between ambient air pollution and anemia within the COPD group (Supplementary Table S11). Most estimates were close to the null. The only nominal association was observed for the 3-month PM_2.5_ average and was inverse (OR = 0.929, 95% CI: 0.863–1.000; *p* = 0.0495). The COPD-with-anemia vs. control-with-anemia comparison showed a pattern broadly similar to the primary COPD analysis, whereas neither the overall anemia analysis nor the within-COPD anemia analysis showed a consistent positive association between ambient air pollution and anemia status.

### 3.3 Polygenic risk score-air pollution interaction analyses

Table 3 shows that most interactions between air pollutants and the lung-function PRS based on FEV_1_/FVC (FEV_1_/FVC PRS) were not significant. For COPD risk, a nominal interaction was observed for 6-month O_3_, with an interaction odds ratio (OR) of 1.009 (95% CI: 1.002–1.015). For continuous FEV_1_/FVC ratio, nominal interactions were observed only for 12-month NO_2_ (*β* = 0.041, 95% CI: 0.003–0.078) and 6-month O_3_ (*β* = −0.070, 95% CI: −0.120 to −0.020). COPD PRS-air pollution interaction analyses are shown in Supplementary Table S12, with no significant interactions identified. Table 4 shows that most interactions between air pollutants and the HGB PRS were not significant for anemia risk in COPD. However, SO_2_ showed nominal positive interactions across all four exposure periods, with interaction ORs ranging from 1.062 to 1.093. For continuous HGB in COPD, inverse interactions with SO_2_ were observed at the 3-, 6-, and 12-month periods, with *β* coefficients ranging from −0.031 to −0.026. These findings suggest that HGB-related genetic susceptibility may modify the association of SO_2_ with both anemia risk and HGB level in COPD. The corresponding findings for binary anemia and continuous HGB provided cross-outcome consistency, whereas the associations across the overlapping SO₂ exposure averages were not considered independent replication. Additional analyses for the RBC, hematocrit, MCV, MCH, and MCHC PRSs are presented in Supplementary Table S13.

**Table 3:** FEV_1_/FVC PRS-air pollution interaction analyses for COPD risk and FEV_1_/FVC ratio.

Exposure variable	Interaction OR (95% CI)^a^	*p*-value	Interaction *β* (95% CI)^a^	*p*-value
	COPD risk		FEV_1_/FVC ratio	
PM_10_				
1-month	1.000 (0.988–1.013)	0.987	0.051 (−0.048–0.150)	0.315
3-month	1.006 (0.992–1.020)	0.393	0.015 (−0.093–0.124)	0.781
6-month	1.010 (0.995–1.026)	0.181	0.003 (−0.112–0.118)	0.958
12-month	1.005 (0.988–1.023)	0.539	0.066 (−0.062–0.193)	0.315
**PM_2.5_**				
1-month	1.011 (0.987–1.036)	0.372	−0.003 (−0.192–0.186)	0.979
3-month	1.020 (0.994–1.048)	0.138	−0.069 (−0.275–0.136)	0.509
6-month	1.024 (0.995–1.054)	0.103	−0.060 (−0.282–0.162)	0.595
12-month	1.012 (0.978–1.047)	0.498	0.088 (−0.162–0.337)	0.491
**NO_2_**				
1-month	0.999 (0.995–1.003)	0.705	0.021 (−0.011–0.053)	0.191
3-month	1.000 (0.996–1.004)	0.998	0.015 (−0.019–0.048)	0.391
6-month	1.000 (0.995–1.004)	0.911	0.017 (−0.018–0.052)	0.349
12-month	0.997 (0.992–1.002)	0.220	**0.041** (0.003–0.078)*	**<0**.**05**
**NO**				
1-month	1.000 (0.996–1.005)	0.924	0.011 (−0.026–0.048)	0.558
3-month	1.001 (0.996–1.006)	0.661	0.005 (−0.036–0.047)	0.806
6-month	1.001 (0.996–1.006)	0.697	0.005 (−0.038–0.047)	0.829
12-month	1.001 (0.995–1.007)	0.729	0.011 (−0.035–0.057)	0.635
**NOx**				
1-month	1.000 (0.997–1.002)	0.861	0.013 (−0.006–0.032)	0.189
3-month	1.000 (0.997–1.003)	0.972	0.011 (−0.010–0.031)	0.309
6-month	1.000 (0.997–1.003)	0.908	0.010 (−0.011–0.031)	0.366
12-month	1.000 (0.997–1.002)	0.743	0.016 (−0.007–0.038)	0.172
**SO_2_**				
1-month	0.992 (0.972–1.012)	0.410	0.125 (−0.031–0.281)	0.116
3-month	0.990 (0.970–1.011)	0.369	0.133 (−0.031–0.297)	0.111
6-month	0.994 (0.972–1.015)	0.561	0.138 (−0.028–0.305)	0.102
12-month	0.993 (0.971–1.015)	0.527	0.154 (−0.013–0.322)	0.070
**CO**				
1-month	1.034 (0.909–1.175)	0.614	0.219 (−0.804–1.243)	0.675
3-month	1.077 (0.938–1.237)	0.290	−0.147 (−1.244–0.951)	0.793
6-month	1.083 (0.936–1.253)	0.286	−0.133 (−1.298–1.033)	0.824
12-month	1.027 (0.880–1.200)	0.733	0.403 (−0.850–1.656)	0.528
**O_3_**				
1-month	1.001 (0.997–1.005)	0.632	−0.016 (−0.044–0.013)	0.281
3-month	1.003 (0.998–1.008)	0.219	−0.034 (−0.072–0.004)	0.075
6-month	**1.009** (1.002–1.015)*	**<0**.**05**	**−0.070** (−0.120 – −0.020)*	**<0**.**05**
12-month	1.006 (0.999–1.014)	0.107	−0.047 (−0.106–0.012)	0.117

COPD, chronic obstructive pulmonary disease; FVC, forced vital capacity; FEV_1_, forced expiratory volume in 1 s; PM_10_, particulate matter with an aerodynamic diameter ≤ 10 µm; PM_2.5_, particulate matter with an aerodynamic diameter ≤ 2.5 µm; NO_2_, nitrogen dioxide; NO, nitric oxide (nitrogen monoxide); NOx, nitrogen oxides; SO_2_, sulfur dioxide; CO, carbon monoxide; O_3_, ozone. Odds ratios (ORs) indicate the risk based on logistic regression models, per 10 µg/m^3^ increase in PM_10_ and PM_2.5_ concentrations, and per 1-unit increase in concentrations of other pollutants. Bold values indicate statistically significant results (*p* < 0.05).

a Adjusted for age, sex, body mass index (BMI), smoking experience, and principal components (PC1-PC10).

* *p* < 0.05.

**Table 4:** HGB PRS-air pollution interaction analyses for anemia risk and HGB in COPD.

Exposure variable	Interaction OR (95% CI)^a^	*p*-value	Interaction β (95% CI)^a^	*p*-value
	Anemia risk in COPD		HGB in COPD	
**PM_10_**				
1-month	1.014 (0.979–1.050)	0.441	−0.003 (−0.016–0.011)	0.690
3-month	1.014 (0.976–1.055)	0.475	−0.001 (−0.016–0.013)	0.866
6-month	1.021 (0.978–1.066)	0.338	−0.007 (−0.023–0.009)	0.413
12-month	1.029 (0.981–1.079)	0.246	−0.014 (−0.032–0.005)	0.148
**PM_2.5_**				
1-month	1.009 (0.945–1.077)	0.793	0.000 (−0.025–0.025)	0.998
3-month	1.011 (0.939–1.089)	0.767	0.003 (−0.025–0.030)	0.858
6-month	1.025 (0.944–1.113)	0.550	−0.008 (−0.040–0.023)	0.595
12-month	1.071 (0.973–1.179)	0.161	−0.030 (−0.067–0.007)	0.109
**NO_2_**				
1-month	1.002 (0.991–1.013)	0.723	0.001 (−0.004–0.005)	0.810
3-month	1.006 (0.994–1.018)	0.366	0.000 (−0.005–0.004)	0.883
6-month	1.009 (0.996–1.021)	0.189	−0.002 (−0.007–0.003)	0.407
12-month	1.013 (0.999–1.027)	0.063	−0.004 (−0.009–0.002)	0.161
**NO**				
1-month	1.006 (0.993–1.019)	0.381	0.000 (−0.005–0.005)	0.972
3-month	1.002 (0.988–1.017)	0.763	0.001 (−0.004–0.007)	0.646
6-month	1.003 (0.989–1.017)	0.650	0.001 (−0.005–0.006)	0.827
12-month	1.008 (0.994–1.022)	0.283	−0.001 (−0.007–0.005)	0.688
**NOx**				
1-month	1.001 (0.994–1.008)	0.738	0.000 (−0.002–0.003)	0.889
3-month	1.004 (0.997–1.011)	0.259	0.000 (−0.003–0.002)	0.718
6-month	1.005 (0.998–1.012)	0.163	−0.001 (−0.004–0.002)	0.431
12-month	1.007 (0.999–1.014)	0.080	−0.002 (−0.005–0.001)	0.244
**SO_2_**				
1-month	**1.062** (1.004–1.124)*	**<0**.**05**	−0.017 (−0.039–0.005)	0.124
3-month	**1.081** (1.018–1.148)*	**<0**.**05**	**−0.026** (−0.050 – −0.003)*	**<0**.**05**
6-month	**1.090** (1.026–1.159)*	**<0**.**05**	**−0.031** (−0.055 – −0.007)*	**<0**.**05**
12-month	**1.093** (1.030–1.160)*	**<0**.**05**	**−0.030** (−0.054 – −0.007)*	**<0**.**05**
**CO**				
1-month	1.135 (0.807–1.595)	0.466	0.022 (−0.111–0.155)	0.746
3-month	1.110 (0.769–1.600)	0.578	0.045 (−0.097–0.187)	0.536
6-month	1.115 (0.762–1.633)	0.574	0.028 (−0.122–0.178)	0.715
12-month	1.233 (0.818–1.860)	0.317	−0.005 (−0.169–0.158)	0.949
**O_3_**				
1-month	1.001 (0.991–1.011)	0.800	−0.001 (−0.005–0.003)	0.684
3-month	1.002 (0.989–1.016)	0.760	0.000 (−0.005–0.006)	0.873
6-month	0.989 (0.970–1.008)	0.244	0.003 (−0.004–0.010)	0.396
12-month	0.989 (0.968–1.009)	0.277	0.002 (−0.006–0.010)	0.626

COPD, chronic obstructive pulmonary disease; HGB, hemoglobin; PM_10_, particulate matter with an aerodynamic diameter ≤ 10 µm; PM_2.5_, particulate matter with an aerodynamic diameter ≤ 2.5 µm; NO_2_, nitrogen dioxide; NO, nitric oxide (nitrogen monoxide); NOx, nitrogen oxides; SO_2_, sulfur dioxide; CO, carbon monoxide; O_3_, ozone. Odds ratios (ORs) indicate the risk based on logistic regression models, per 10 µg/m^3^ increase in PM_10_ and PM_2.5_ concentrations, and per 1-unit increase in concentrations of other pollutants. Bold values indicate statistically significant results (*p* < 0.05).

a Adjusted for age, sex, body mass index (BMI), smoking experience, and principal components (PC1-PC10).

* *p* < 0.05.

### 3.4 GWAS and SNP-air pollution interaction analyses for COPD

Supplementary Figure S1 and Table S14 show the GWAS results for COPD. Two lead loci were identified, including rs4808295-A in *ZNF100*, which was associated with lower COPD risk (OR = 0.865, *p* = 1.63 × 10^−10^) and rs138998202-T in *LOC124903865* on chromosome 1, which was associated with higher COPD risk (OR = 1.434, *p* = 1.91 × 10^−8^). The genomic inflation factor was modest (*λ*gc = 1.046). In the sensitivity GWAS, rs4808295 remained stable whereas the signal in *LOC124903865* was not retained (Supplementary Figure S2 and Table S15), suggesting that *ZNF100* was the locus supported by sensitivity analysis in this dataset. Table 5 shows selective evidence of interaction between candidate COPD-associated loci and air pollution exposure. For rs4808295-A in *ZNF100*, nominal positive interactions were observed with PM_2.5_ at 6 months and 12 months, with additional nominal interactions for NO, NOx, SO_2_, and CO, whereas 1-month O_3_ showed a nominal inverse interaction. For rs138998202-T in *LOC124903865*, nominal inverse interactions were observed with NO_2_ at 1 and 12 months, while 3-month O_3_ showed a nominal positive interaction. Overall, these findings identified selective SNP-air pollution interaction patterns, particularly for *ZNF100*, which were considered exploratory.

**Table 5:** GWAS-informed candidate SNP-air pollution interaction analyses for COPD risk.

Exposure variable	Interaction OR (95% CI)^a^	*p*-value	Interaction OR (95% CI)^a^	*p*-value
	rs4808295-A in *ZNF100*		rs138998202-T in *LOC124903865*	
**PM_10_**				
1-month	0.995 (0.974–1.017)	0.672	0.995 (0.933–1.061)	0.878
3-month	1.005 (0.982–1.029)	0.663	1.025 (0.956–1.099)	0.487
6-month	1.023 (0.997–1.050)	0.079	1.040 (0.965–1.121)	0.304
12-month	1.030 (1.000–1.061)	0.053	1.022 (0.936–1.116)	0.630
**PM_2.5_**				
1-month	1.001 (0.959–1.044)	0.977	0.963 (0.853–1.088)	0.550
3-month	1.021 (0.976–1.069)	0.370	1.043 (0.912–1.193)	0.536
6-month	**1.057** (1.006–1.111)*	**<0**.**05**	1.068 (0.925–1.233)	0.368
12-month	**1.089** (1.027–1.154)*	**<0**.**05**	1.050 (0.882–1.250)	0.585
**NO_2_**				
1-month	1.000 (0.993–1.007)	0.969	**0.973** (0.954–0.993)*	**<0**.**05**
3-month	1.002 (0.995–1.010)	0.567	0.980 (0.959–1.001)	0.060
6-month	1.007 (0.999–1.015)	0.105	0.985 (0.964–1.007)	0.186
12-month	1.008 (1.000–1.017)	0.063	**0.973** (0.950–0.997)*	**<0**.**05**
**NO**				
1-month	1.007 (0.999–1.015)	0.078	0.980 (0.958–1.002)	0.080
3-month	1.007 (0.998–1.016)	0.140	0.977 (0.953–1.001)	0.065
6-month	1.008 (0.999–1.018)	0.078	0.981 (0.957–1.006)	0.135
12-month	**1.013** (1.003–1.023)*	**<0**.**05**	0.982 (0.955–1.010)	0.202
**NOx**				
1-month	1.002 (0.998–1.006)	0.384	0.988 (0.975–1.000)	0.051
3-month	1.002 (0.998–1.007)	0.267	0.988 (0.976–1.001)	0.064
6-month	**1.005** (1.000–1.009)*	**<0**.**05**	0.991 (0.978–1.004)	0.156
12-month	**1.005** (1.001–1.010)*	**<0**.**05**	0.987 (0.974–1.001)	0.066
**SO_2_**				
1-month	**1.037** (1.002–1.074)*	**<0**.**05**	0.984 (0.890–1.088)	0.757
3-month	1.032 (0.996–1.070)	0.078	0.981 (0.880–1.094)	0.731
6-month	1.026 (0.989–1.064)	0.167	0.949 (0.848–1.061)	0.357
12-month	1.032 (0.994–1.071)	0.097	0.932 (0.834–1.042)	0.216
**CO**				
1-month	1.060 (0.853–1.317)	0.600	0.591 (0.314–1.112)	0.103
3-month	1.098 (0.869–1.389)	0.433	0.710 (0.360–1.402)	0.324
6-month	1.273 (0.994–1.630)	0.056	0.785 (0.387–1.595)	0.504
12-month	**1.352** (1.039–1.761)*	**<0**.**05**	0.662 (0.307–1.424)	0.291
**O_3_**				
1-month	**0.992** (0.986–0.999)*	**<0**.**05**	1.012 (0.994–1.030)	0.184
3-month	0.992 (0.983–1.000)	0.057	**1.026** (1.001–1.051)*	**<0**.**05**
6-month	0.994 (0.983–1.005)	0.294	1.014 (0.981–1.048)	0.425
12-month	0.993 (0.980–1.006)	0.307	1.021 (0.983–1.060)	0.286

PM_10_, particulate matter with an aerodynamic diameter ≤ 10 µm; PM_2.5_, particulate matter with an aerodynamic diameter ≤ 2.5 µm; NO_2_, nitrogen dioxide; NO, nitric oxide (nitrogen monoxide); NOx, nitrogen oxides; SO_2_, sulfur dioxide; CO, carbon monoxide; O_3_, ozone. Odds ratios (ORs) indicate the risk based on logistic regression models, per 10 µg/m^3^ increase in PM_10_ and PM_2.5_ concentrations, and per 1-unit increase in concentrations of other pollutants. Bold values indicate statistically significant results (*p* < 0.05).

a Adjusted for age, sex, body mass index (BMI), smoking experience, and principal components (PC1-PC10).

* *p* < 0.05.

### 3.5 GWAS and SNP-air pollution interaction analyses for anemia in COPD

Supplementary Figure S3 and Table S16 summarize the GWAS results for anemia within the COPD group. Although three genome-wide significant signals were identified in the primary analysis, none of the corresponding lead variants remained stable in the sensitivity GWAS (Supplementary Figure S4, Table S17). These loci were therefore considered exploratory and hypothesis-generating, with detailed locus-level results provided in the Supplementary Information. Table 6 shows limited evidence of interaction between candidate anemia-associated loci and air pollutants in relation to anemia risk in COPD. No significant interactions were observed for rs375498857-A in *PGAP6* or rs183154008-C in *OR52E1*. In contrast, rs11038031-G in *OR52H1/OR52V1P* showed a nominal inverse interaction with 1-month O_3_ exposure (OR = 0.922, 95% CI: 0.853–0.996). Overall, these findings suggest that gene-environment interaction signals for anemia risk in COPD were limited and restricted to specific locus-pollutant combinations.

**Table 6:** GWAS-informed candidate SNP-air pollutant interaction analyses for anemia risk in COPD.

Exposure variable	Interaction OR (95% CI)^a^	*p*-value	Interaction OR (95% CI)^a^	*p*-value	Interaction OR (95% CI)^a^	*p*-value
	rs375498857-A in *PGAP6*		rs183154008-C in *OR52E1*		rs11038031-G in *OR52H1/OR52V1P*	
**PM_10_**						
1-month	1.005 (0.872–1.157)	0.950	0.961 (0.797–1.160)	0.682	0.870 (0.655–1.156)	0.337
3-month	0.970 (0.827–1.139)	0.713	0.953 (0.757–1.200)	0.682	0.868 (0.638–1.182)	0.370
6-month	0.959 (0.802–1.148)	0.651	0.905 (0.684–1.198)	0.486	0.858 (0.627–1.175)	0.341
12-month	0.928 (0.757–1.137)	0.470	1.040 (0.763–1.419)	0.804	0.878 (0.610–1.263)	0.483
**PM_2.5_**						
1-month	1.001 (0.764–1.311)	0.994	1.004 (0.694–1.454)	0.983	0.884 (0.541–1.445)	0.622
3-month	0.952 (0.704–1.286)	0.747	0.937 (0.616–1.424)	0.759	0.899 (0.536–1.509)	0.688
6-month	0.839 (0.591–1.191)	0.326	0.816 (0.485–1.373)	0.444	0.796 (0.435–1.457)	0.459
12-month	0.748 (0.489–1.144)	0.181	1.155 (0.628–2.125)	0.643	0.831 (0.385–1.795)	0.638
**NO_2_**						
1-month	1.001 (0.953–1.051)	0.967	1.000 (0.938–1.066)	1.000	1.023 (0.934–1.120)	0.627
3-month	0.994 (0.944–1.047)	0.819	0.981 (0.912–1.055)	0.609	1.014 (0.922–1.116)	0.768
6-month	0.994 (0.939–1.052)	0.839	0.959 (0.880–1.044)	0.333	0.984 (0.881–1.100)	0.782
12-month	0.991 (0.930–1.057)	0.791	0.986 (0.901–1.080)	0.765	0.995 (0.878–1.126)	0.931
**NO**						
1-month	1.008 (0.949–1.070)	0.806	0.945 (0.853–1.048)	0.283	1.061 (0.940–1.197)	0.340
3-month	1.009 (0.948–1.074)	0.776	0.939 (0.843–1.047)	0.257	1.041 (0.910–1.189)	0.559
6-month	1.006 (0.945–1.070)	0.858	0.935 (0.836–1.044)	0.233	0.999 (0.861–1.159)	0.989
12-month	0.998 (0.931–1.069)	0.948	0.967 (0.878–1.064)	0.489	0.985 (0.849–1.143)	0.842
**NOx**						
1-month	0.998 (0.967–1.030)	0.916	1.002 (0.969–1.037)	0.887	1.009 (0.953–1.067)	0.762
3-month	0.996 (0.964–1.030)	0.816	0.985 (0.941–1.030)	0.503	1.000 (0.941–1.063)	0.989
6-month	0.997 (0.962–1.032)	0.848	0.980 (0.933–1.029)	0.419	0.982 (0.915–1.053)	0.609
12-month	0.994 (0.958–1.031)	0.743	0.997 (0.950–1.045)	0.888	0.989 (0.919–1.063)	0.758
**SO_2_**						
1-month	1.032 (0.826–1.290)	0.782	1.066 (0.800–1.421)	0.663	1.157 (0.829–1.617)	0.391
3-month	0.985 (0.767–1.265)	0.906	1.091 (0.801–1.487)	0.579	1.157 (0.815–1.641)	0.415
6-month	0.962 (0.744–1.245)	0.768	1.062 (0.756–1.492)	0.730	1.070 (0.737–1.554)	0.722
12-month	0.945 (0.732–1.220)	0.665	1.093 (0.762–1.569)	0.629	1.065 (0.698–1.626)	0.770
**CO**						
1-month	1.071 (0.218–5.271)	0.933	0.828 (0.118–5.822)	0.850	1.490 (0.074–29.994)	0.795
3-month	1.099 (0.199–6.054)	0.914	0.539 (0.062–4.717)	0.577	1.561 (0.070–34.602)	0.778
6-month	1.198 (0.192–7.492)	0.847	0.420 (0.041–4.275)	0.464	0.862 (0.026–28.043)	0.933
12-month	1.082 (0.149–7.838)	0.938	0.586 (0.052–6.608)	0.666	0.817 (0.020–32.823)	0.914
**O_3_**						
1-month	1.000 (0.955–1.047)	0.994	0.985 (0.929–1.045)	0.620	**0.922** (0.853–0.996)*	**<0**.**05**
3-month	0.996 (0.936–1.060)	0.902	0.973 (0.898–1.054)	0.496	0.924 (0.834–1.025)	0.135
6-month	0.974 (0.888–1.069)	0.583	0.940 (0.832–1.061)	0.314	0.977 (0.829–1.152)	0.783
12-month	0.961 (0.870–1.062)	0.436	0.971 (0.852–1.107)	0.660	0.969 (0.810–1.160)	0.735

PM_10_, particulate matter with an aerodynamic diameter ≤10 µm; PM_2.5_, particulate matter with an aerodynamic diameter ≤2.5 µm; NO_2_, nitrogen dioxide; NO, nitric oxide (nitrogen monoxide); NOx, nitrogen oxides; SO_2_, sulfur dioxide; CO, carbon monoxide; O_3_, ozone. Odds ratios (ORs) indicate the risk based on logistic regression models, per 10 µg/m^3^ increase in PM_10_ and PM_2.5_ concentrations, and per 1-unit increase in concentrations of other pollutants. Bold values indicate statistically significant results (*p* < 0.05).

a Adjusted for age, sex, body mass index (BMI), smoking experience, and principal components (PC1-PC10).

* *p* < 0.05.

## 4 Discussion

We showed that higher air pollution exposure was associated with higher COPD risk. For PM_2.5_, each 10 µg/m^3^ increase was associated with COPD. Positive associations were also observed in the COPD-with-anemia vs. control-with-anemia comparison, whereas the overall anemia analysis showed no consistent positive pattern. The most consistent gene-environment finding involved the HGB PRS and SO_2_, while rs4808295-A in *ZNF100* was the COPD-associated locus supported by sensitivity analysis. Together, these findings support associations of ambient air pollution with COPD and identify selected genetic susceptibility signals for further investigation.

The positive associations observed for particulate and combustion-related pollutants are consistent with previous epidemiological evidence showing associations between air pollution, COPD development, and impaired lung function. Previous epidemiological evidence has reported an 18% higher risk of incident COPD per 10 µg/m^3^ increase in PM_2.5_ (95% CI: 1.13–1.23) (6). A longitudinal Taiwanese cohort reported a hazard ratio (HR) of 0.88 (95% CI: 0.83–0.93) per 5 µg/m^3^ decrease in PM_2.5_ change, corresponding approximately to an HR of 1.29 (95% CI: 1.16–1.45) per 10 µg/m³ increase when rescaled under a log-linear assumption (7). The present 12-month estimate (OR = 1.642, 95% CI: 1.588–1.699 per 10 µg/m^3^) was greater than these previous estimates. However, direct comparison should consider differences in the epidemiologic contrasts: the Taiwanese cohort evaluated longitudinal changes in PM_2.5_ in relation to incident COPD, whereas the present study evaluated pre-recruitment residential exposure levels in a cross-sectional case-control comparison. Differences in COPD ascertainment, study population, and exposure assessment may also contribute to the observed difference in effect magnitude. Importantly, restricting the analysis to spirometry-defined airflow obstruction produced estimates closely corresponding to those of the primary analysis, indicating that the observed associations were not driven by the small proportion of cases identified solely through self-report. In addition, broader averaging periods may reduce short-term temporal variability in the residential exposure estimate. Accordingly, differences in effect magnitude across the overlapping averaging periods should not be interpreted as evidence of cumulative exposure or a specific etiologic latency period. PM_2.5_ may contribute through deep airway penetration, oxidative stress, inflammation, and airway remodeling, whereas the observed correlations among SO_2_, NOx, CO, and other pollutants indicate that associations with individual pollutants may also reflect shared combustion-related exposure mixtures (34, 35). The practical relevance of these associations should be interpreted in relation to the exposure increment. PM_10_ and PM_2.5_ estimates were expressed per 10 µg/m^3^ increase, whereas gaseous pollutants were modeled per 1-unit increase; therefore, ORs close to 1 for gaseous pollutants represent comparatively small exposure increments. Although modest at the individual level, such associations may remain relevant at the population level because ambient air pollution is widespread. The findings were therefore interpreted according to effect magnitude and confidence intervals rather than statistical significance alone.

In the COPD-with-anemia vs. control-with-anemia comparison, each 10 µg/m^3^ increase in PM_2.5_ was associated with ORs of 1.225 (95% CI: 1.137–1.319) at 1 month and 1.679 (95% CI: 1.517–1.859) at 12 months. However, because anemia was present in both comparison groups, these estimates primarily represent a COPD contrast among participants with anemia rather than an anemia-specific pollution effect. Consistent with this interpretation, the within-COPD anemia analysis showed no consistent positive association between ambient air pollution and anemia status. The only nominal association was observed for the 3-month PM_2.5_ average and was inverse (OR = 0.929, 95% CI: 0.863–1.000; *p* = 0.0495). Similarly, the overall anemia analysis showed inverse PM_2.5_ associations at several averaging periods and no consistent positive pattern for gaseous pollutants. These findings therefore do not support a general positive association between ambient air pollution and anemia in this population. Previous studies have reported associations of particulate and traffic-related air pollution with lower HGB concentrations and higher anemia prevalence (11, 36). The contrasting findings in the present study may reflect differences in population characteristics, anemia definition, exposure assessment, or residual confounding. Because anemia was defined solely by hemoglobin concentration, the present data do not distinguish anemia related to iron deficiency, inflammation, nutritional deficiency, occult blood loss, or other causes. The findings should therefore be interpreted as associations with anemia status rather than with a specific anemia subtype. The inverse estimates should not be interpreted as evidence of a protective effect and warrant further investigation in studies designed specifically to evaluate anemia incidence and etiology. In contrast, O_3_ showed inverse associations at selected exposure periods. In the COPD analysis, the association was inverse at 6 months (OR = 0.982, 95% CI: 0.976–0.989) but null at 12 months (OR = 1.003, 95% CI: 0.996–1.011). This pattern should not be interpreted as evidence of a protective effect of O_3_. Freshly emitted NOx from traffic and other combustion sources can reduce local O_3_ concentrations through titration, resulting in lower O_3_ levels in areas with higher primary pollutant concentrations (37). In addition, O_3_ may represent a different spatial and temporal pollution contrast because its concentrations are influenced by photochemical production, atmospheric transport, and meteorological conditions (38). Differences across exposure periods may also reflect variation in the temporal exposure metric, spatial representativeness, and correlations between O_3_ and co-pollutants (39). Thus, the inverse O_3_ estimates are better interpreted as context-dependent exposure associations rather than evidence of a direct beneficial effect.

The most consistent gene-environment finding involved the HGB PRS and SO_2_. For anemia risk in COPD, the interaction OR increased from 1.062 (95% CI: 1.004–1.124) at 1 month to 1.093 (95% CI: 1.030–1.160) at 12 months, with nominal interactions also observed at 3 and 6 months. For continuous HGB, inverse interactions were observed at 3 months (*β* = −0.026, 95% CI: −0.050 to −0.003), 6 months (*β* = −0.031, 95% CI: −0.055 to −0.007), and 12 months (*β* = −0.030, 95% CI: −0.054 to −0.007). SO_2_ is a respiratory toxicant and an indicator of combustion-related exposure that may induce systemic oxidative stress, including oxidative effects on erythrocytes (40, 41). The HGB PRS-SO_2_ findings suggest that inherited susceptibility related to lower HGB may contribute to heterogeneity in hematologic responses to residential ambient SO_2_ or correlated combustion-related exposure in COPD, rather than a uniform positive association across all participants with COPD. Accordingly, these findings should not be interpreted as evidence of an isolated pollutant-specific effect. The predominance of null findings for the other PRSs did not support broad genetic modification of air pollution associations. Gene-environment interaction analyses generally require greater statistical power than main-effect analyses; therefore, some null interactions may reflect limited power, whereas isolated nominal findings may represent chance associations. The corresponding HGB PRS-SO_2_ findings for binary anemia and continuous HGB provided cross-outcome consistency. However, because the SO₂ averaging periods were strongly correlated (*r* = 0.898–0.970), recurrence across these periods was not considered independent replication. This distinguishes the cross-outcome pattern from isolated nominal interactions, which were interpreted as exploratory and require independent replication.

Among the COPD GWAS findings, rs4808295-A in *ZNF100* was associated with lower COPD risk (OR = 0.865, *p* = 1.63 × 10^−10^) and was retained in the sensitivity analysis, providing the strongest single-variant signal in this dataset. Zinc finger proteins are transcriptional regulators implicated in COPD-related and inflammatory pathways, supporting further investigation of *ZNF100* in pollution-related susceptibility (42–45). In contrast, none of the lead loci from the anemia-in-COPD GWAS remained stable in the sensitivity analysis. The primary signals in *PGAP6*, near *OR52E1*, and in *OR52H1/OR52V1P* should therefore be regarded as exploratory and hypothesis-generating. Although selected loci have previously been associated with hematologic traits, such observations provide biological context rather than replication of the present findings (46–49). The largely null candidate SNP-air pollution interaction results further support a cautious interpretation of these loci. Independent replication will be required before they can be considered established susceptibility markers (50, 51).

This study has several strengths. First, it included a large age- and sex-matched Taiwan Biobank sample of 7,566 COPD cases and 22,698 controls, providing substantial statistical information for environmental and genetic analyses. Second, residential exposure to eight ambient air pollutants was evaluated across four prespecified exposure-averaging periods, allowing associations to be compared across complementary exposure summaries. Third, the study integrated externally derived PRSs, GWAS, and gene-environment interaction analyses within the same analytical framework. East Asian summary statistics were used for hematologic PRSs when available, and sensitivity GWAS was conducted to assess the stability of lead loci. Finally, both binary phenotypes and continuous spirometric and hematologic traits were evaluated, enabling comparison across related outcomes.

This study has several limitations. The cross-sectional case-control design limits temporal and causal inference, and monitoring-station-based residential exposure estimates may not fully capture individual exposure patterns or fine-scale spatial variability; participants assigned to the same monitoring station may also share similar exposure estimates. Because station-assignment identifiers were not retained in the participant-level analytic dataset, station-level dependence in the variance estimates could not be directly evaluated. The 1-, 3-, 6-, and 12-month exposure averages were nested and correlated; therefore, differences across averaging periods were not interpreted as independent temporal effects. Although the COPD definition incorporated both spirometric and self-reported information, 96.1% of cases met the spirometry-defined airflow-obstruction criterion, and the spirometry-restricted sensitivity analysis produced closely comparable estimates. The prespecified single-pollutant models may reflect correlated pollution mixtures rather than independent pollutant-specific effects. No formal multiplicity correction was applied outside the GWAS analyses; therefore, nominal interaction findings should be interpreted as exploratory. The COPD-with-anemia vs. control-with-anemia comparison represents a COPD contrast among participants with anemia rather than an anemia-specific effect, while anemia itself was defined using HGB thresholds without biomarkers permitting etiologic subtyping. Although additional adjustment for smoking duration and intensity produced materially unchanged estimates, residual confounding from tobacco exposure, occupational and socioeconomic conditions, indoor pollution, comorbidities, and nutritional factors cannot be excluded.

## 5 Conclusions

Air pollution exposure was associated with COPD across the evaluated pre-recruitment exposure-averaging periods. Positive associations were also observed when COPD cases with anemia were compared with controls with anemia; however, the within-COPD anemia analysis showed no consistent positive association between ambient air pollution and anemia status. The nominal HGB PRS-SO_2_ interaction pattern and the sensitivity-supported *ZNF100* association identified genetic susceptibility signals for further investigation. These findings support an integrated environmental and genetic framework for understanding COPD and related hematologic variation.

## Data Availability

The data analyzed in this study is subject to the following licenses/restrictions: The Taiwan Biobank individual-level genotype and phenotype data are restricted due to institutional and ethical regulations. They are not publicly available and can only be accessed through application and approval by the Taiwan Biobank. Requests to access these datasets should be directed to Taiwan Biobank Data Release Group, biobank@gate.sinica.edu.tw.
