# Supplementary material for: Association of air pollution with chronic obstructive pulmonary disease: anemia-related outcomes and exploratory genetic susceptibility analyses
Source: Front Med (Lausanne). 2026 Sep 8;13:1925502. doi: 10.3389/fmed.2026.1925502 (PMC13593447; doi:10.3389/fmed.2026.1925502)
Supplement: Supplementary file 1 [file Datasheet1.docx]

**Supplementary Information**

**Association of Air Pollution With Chronic Obstructive Pulmonary Disease: Anemia-Related Outcomes and Exploratory Genetic Susceptibility Analyses**

Chi-Thien Dinh^1,2^ (MD, MSc), Yueh-Lun Lee^3^ (MD, PhD), Xuan Ngoc Tran^1^ (MD, MSc), Kang-Yun Lee^4,5,6^ (MD, PhD), Kian Fan Chung^7,8^ (MD, DSc), Jer-Hwa Chang^9,10*^ (MD, MSc), Hsiao-Chi Chuang^9,10,11,12*^ (PhD)

^1^International Ph.D. Program in Medicine, College of Medicine, Taipei Medical University, Taipei, Taiwan

^2^Department of Internal Medicine, Faculty of Medicine, Can Tho University of Medicine and Pharmacy, Can Tho, Vietnam

^3^Department of Microbiology and Immunology, College of Medicine, Taipei Medical University, Taipei, Taiwan

^4^Division of Pulmonary Medicine, Department of Internal Medicine, Shuang Ho Hospital, Taipei Medical University, New Taipei City, Taiwan

^5^Graduate Institute of Clinical Medicine, College of Medicine, Taipei Medical University, Taipei, Taiwan

^6^Division of Pulmonary Medicine, Department of Internal Medicine, School of Medicine, College of Medicine, Taipei Medical University, Taipei, Taiwan

^7^National Heart and Lung Institute, Imperial College London, London SW3, UK

^8^Royal Brompton & Harefield Hospitals, Guy’s and St Thomas’ NHS Foundation Trust, London SW3, UK

^9^School of Respiratory Therapy, College of Medicine, Taipei Medical University, Taipei, Taiwan

^10^Division of Pulmonary Medicine, Department of Internal Medicine, Wan Fang Hospital, Taipei Medical University, Taipei, Taiwan

^11^Division of Pulmonary Medicine, Department of Internal Medicine, Shuang Ho Hospital, Taipei Medical University, New Taipei City, Taiwan

^12^Cell Physiology and Molecular Image Research Center, Wan Fang Hospital, Taipei Medical University, Taipei, Taiwan

***Corresponding Authors**

*Jer-Hwa Chang, MD, MSc*

School of Respiratory Therapy, College of Medicine, Taipei Medical University, 250 Wuxing Street, Taipei 11031, Taiwan.

Telephone: +886-2-27361661 ext. 27483. Fax: +886-2-27391143. E-mail: [jerhwa@tmu.edu.tw](mailto:jerhwa@tmu.edu.tw)

*Hsiao-Chi Chuang, PhD*

Inhalation Toxicology Research Lab (ITRL), School of Respiratory Therapy, College of Medicine, Taipei Medical University, 250 Wuxing Street, Taipei 11031, Taiwan.

Telephone: +886-2-27361661 ext. 27483. Fax: +886-2-27391143. E-mail: [chuanghc@tmu.edu.tw](mailto:chuanghc@tmu.edu.tw)

*S1. Participant selection*

Baseline Taiwan Biobank (TWB) participants were screened for eligibility. Adults aged 20 years or older were included to reduce under-ascertainment of younger individuals with airflow obstruction, as chronic obstructive pulmonary disease (COPD) may be under-recognized in younger adults and is not defined by age alone in the Global Initiative for Chronic Obstructive Lung Disease (GOLD) framework [1]. Participants were required to have complete data on variables necessary for phenotype definition, exposure assessment, and covariate adjustment, including spirometric indices, hematological parameters, anthropometric variables, smoking-related variables, air pollution exposure estimates, genotype linkage, and principal components. Individuals with a history of cancer, renal failure, or asthma were excluded because these conditions could influence hematological traits or complicate COPD classification. Only baseline records were retained because genotype data were available only at baseline. Genotype data were obtained from the TWB combined high-confidence release. According to the release documentation, quality control procedures for kinship and principal component analysis (PCA) generation included filtering on sample missingness, single-nucleotide polymorphism (SNP) missingness, heterozygosity outliers, and minor allele frequency. For PCA generation, additional exclusions were applied for duplicate or monozygotic twin samples, non-East Asian individuals, variants deviating from Hardy-Weinberg equilibrium, and variants located in long-range linkage disequilibrium regions before deriving PC1-PC20. These TWB-derived principal components were used to control for population structure in all genetic analyses.

*S2. Coarsened exact matching procedure*

To improve baseline comparability between COPD cases and controls, coarsened exact matching was implemented using age and sex. Age was grouped into decade-based categories: 20-29, 30-39, 40-49, 50-59, 60-69, 70-79, 80-89, and ≥90 years. Exact matching was then performed on sex. Only strata containing both COPD cases and controls were retained. Within each matched stratum, controls were randomly sampled without replacement at a 1:3 ratio. This ratio was selected to increase control representation while preserving adequate case representation and statistical efficiency for downstream analyses. The resulting coarsened exact matching (CEM)-matched sample served as the common analytic dataset for environmental association analyses, subgroup analyses, genome-wide association analyses, polygenic risk score (PRS) analyses, and interaction models.

*S3. PRS construction and outcome-related interaction analyses*

To avoid sample overlap and potential inflation of PRS estimates, polygenic scores were derived from external resources rather than from the internal genome-wide association study (GWAS) results of the present study [2]. For COPD risk analyses, published scoring files were obtained from the Polygenic Score Catalog. A lung-function PRS was based on PGS000210 for ratio of forced expiratory volume in 1 second to forced vital capacity (FEV_1_/FVC) [3]. The COPD PRS was based on PGS001332, which was developed using snpnet, mapped to GRCh37, and trained in 269,704 UK Biobank White British participants, including 7,674 COPD cases and 262,030 controls [4]. Both scores were calculated in the TWB target dataset using PLINK v2.0, merged into the matched analytic sample by FID and IID, and standardized as z-scores prior to downstream analyses. For anemia-related interaction analyses, direct anemia diagnostic scores were not used. Instead, hematologic PRS were constructed from external summary statistics derived from BioBank Japan/PheWeb, selected because their East Asian ancestry more closely matched the TWB target population [5]. Trait-specific PRS were generated for hemoglobin (HGB), red blood cell count (RBC), hematocrit, mean corpuscular volume (MCV), mean corpuscular hemoglobin (MCH), and mean corpuscular hemoglobin concentration (MCHC) using PRSice v2.3.5 under the clumping-and-thresholding (C+T) framework (clump-kb = 250, clump-r2 = 0.1, and clump-p = 1). For each hematologic trait, the primary PRS was defined as the full clumped score at *P* ≤ 1 to avoid threshold tuning on the target dataset [2]. These scores were then calculated in the TWB target genotype dataset, merged into the matched analytic sample, and standardized as z-scores.

For COPD-related PRS analyses, the interaction analysis tested whether the association between air pollution exposure and COPD differed according to FEV_1_/FVC PRS. An interaction analysis was also conducted with COPD polygenic risk. When spirometric ratio data were available, additional validation and exploratory analyses examined the association of the FEV_1_/FVC PRS with measured FEV_1_/FVC and tested whether air pollution exposure and the FEV_1_/FVC PRS jointly influenced this spirometric trait. For anemia-related analyses among participants with COPD, PRS-based interaction analyses tested whether the association between air pollution exposure and anemia differed according to hematologic genetic susceptibility. Trait-specific PRSs were evaluated for HGB, RBC, hematocrit, MCV, MCH, and MCHC. Validation analyses assessed whether each PRS was associated with its corresponding hematologic phenotype, and exploratory mechanistic analyses further examined whether air pollution exposure and the corresponding PRS jointly influenced the relevant continuous hematologic trait.

*S4. Genome-wide association analyses workflow*

Primary genome-wide association analyses were performed using PLINK v1.9 under an additive logistic regression model adjusted for age, sex, body mass index (BMI), smoking experience, and principal components 1-10 (PC1-PC10). Two principal GWAS analyses were conducted: one for COPD risk in the matched case–control sample and one for anemia risk within the COPD subgroup. The primary GWAS was run on the TWB combined high-confidence genotype dataset without additional variant-level filters beyond the TWB release and analytic sample restrictions. A sensitivity GWAS applied additional variant-level quality control filters of minor allele frequency (MAF) ≥0.05, genotype missingness ≤0.02, and Hardy–Weinberg equilibrium *P* ≥ 1×10^-6^ (mid-p) to restrict the analysis to common variants with low missingness and Hardy–Weinberg equilibrium support.

Genome-wide significance was defined as *P* <5×10^-8^. Variants with *P* <1×10^-6^ were considered suggestive, and variants with *P* <1×10^-5^ were considered exploratory [6]. To identify approximately independent loci, linkage disequilibrium clumping was performed in PLINK using clump-kb = 250, clump-r2 = 0.1, and clump-p2 = 0.05, with index variant thresholds specified according to the analysis objective. Manhattan plots, quantile-quantile plots, and genomic inflation factors (λ) were used to evaluate GWAS results. Lead loci were further annotated using dbSNP, Ensembl, and the GWAS Catalog to identify genomic context, nearest genes, and previously reported trait associations.

**Table S1. Availability of monthly air pollution monitoring data by pollutant and calendar year, 2012–2019**

| **Pollutant** | **Year** | **Stations represented** | **Observed station-months** | **Expected station-months** | **Monthly completeness (%)** | **Complete stations, n (%)** |
| --- | --- | --- | --- | --- | --- | --- |
| PM_10_ | 2012 | 76 | 909 | 912 | 99.67 | 74 (97.37) |
| PM_10_ | 2013 | 76 | 910 | 912 | 99.78 | 75 (98.68) |
| PM_10_ | 2014 | 76 | 912 | 912 | 100 | 76 (100) |
| PM_10_ | 2015 | 76 | 912 | 912 | 100 | 76 (100) |
| PM_10_ | 2016 | 76 | 912 | 912 | 100 | 76 (100) |
| PM_10_ | 2017 | 76 | 912 | 912 | 100 | 76 (100) |
| PM_10_ | 2018 | 77 | 924 | 924 | 100 | 77 (100) |
| PM_10_ | 2019 | 77 | 924 | 924 | 100 | 77 (100) |
| PM_2.5_ | 2012 | 76 | 907 | 912 | 99.45 | 72 (94.74) |
| PM_2.5_ | 2013 | 76 | 910 | 912 | 99.78 | 75 (98.68) |
| PM_2.5_ | 2014 | 76 | 912 | 912 | 100 | 76 (100) |
| PM_2.5_ | 2015 | 76 | 911 | 912 | 99.89 | 75 (98.68) |
| PM_2.5_ | 2016 | 76 | 912 | 912 | 100 | 76 (100) |
| PM_2.5_ | 2017 | 76 | 910 | 912 | 99.78 | 75 (98.68) |
| PM_2.5_ | 2018 | 77 | 924 | 924 | 100 | 77 (100) |
| PM_2.5_ | 2019 | 77 | 924 | 924 | 100 | 77 (100) |
| NO_2_ | 2012 | 76 | 909 | 912 | 99.67 | 74 (97.37) |
| NO_2_ | 2013 | 76 | 911 | 912 | 99.89 | 75 (98.68) |
| NO_2_ | 2014 | 76 | 912 | 912 | 100 | 76 (100) |
| NO_2_ | 2015 | 76 | 912 | 912 | 100 | 76 (100) |
| NO_2_ | 2016 | 76 | 912 | 912 | 100 | 76 (100) |
| NO_2_ | 2017 | 76 | 912 | 912 | 100 | 76 (100) |
| NO_2_ | 2018 | 77 | 924 | 924 | 100 | 77 (100) |
| NO_2_ | 2019 | 77 | 917 | 924 | 99.24 | 76 (98.7) |
| NO | 2012 | 76 | 909 | 912 | 99.67 | 74 (97.37) |
| NO | 2013 | 76 | 911 | 912 | 99.89 | 75 (98.68) |
| NO | 2014 | 76 | 912 | 912 | 100 | 76 (100) |
| NO | 2015 | 76 | 912 | 912 | 100 | 76 (100) |
| NO | 2016 | 76 | 912 | 912 | 100 | 76 (100) |
| NO | 2017 | 76 | 912 | 912 | 100 | 76 (100) |
| NO | 2018 | 76 | 912 | 912 | 100 | 76 (100) |
| NO | 2019 | 77 | 918 | 924 | 99.35 | 76 (98.7) |
| NOx | 2012 | 76 | 909 | 912 | 99.67 | 74 (97.37) |
| NOx | 2013 | 76 | 911 | 912 | 99.89 | 75 (98.68) |
| NOx | 2014 | 76 | 912 | 912 | 100 | 76 (100) |
| NOx | 2015 | 76 | 912 | 912 | 100 | 76 (100) |
| NOx | 2016 | 76 | 912 | 912 | 100 | 76 (100) |
| NOx | 2017 | 76 | 912 | 912 | 100 | 76 (100) |
| NOx | 2018 | 77 | 924 | 924 | 100 | 77 (100) |
| NOx | 2019 | 77 | 917 | 924 | 99.24 | 76 (98.7) |
| SO_2_ | 2012 | 76 | 909 | 912 | 99.67 | 74 (97.37) |
| SO_2_ | 2013 | 76 | 910 | 912 | 99.78 | 75 (98.68) |
| SO_2_ | 2014 | 76 | 912 | 912 | 100 | 76 (100) |
| SO_2_ | 2015 | 76 | 911 | 912 | 99.89 | 75 (98.68) |
| SO_2_ | 2016 | 76 | 912 | 912 | 100 | 76 (100) |
| SO_2_ | 2017 | 76 | 912 | 912 | 100 | 76 (100) |
| SO_2_ | 2018 | 77 | 924 | 924 | 100 | 77 (100) |
| SO_2_ | 2019 | 77 | 924 | 924 | 100 | 77 (100) |
| CO | 2012 | 75 | 897 | 900 | 99.67 | 73 (97.33) |
| CO | 2013 | 75 | 898 | 900 | 99.78 | 74 (98.67) |
| CO | 2014 | 75 | 900 | 900 | 100 | 75 (100) |
| CO | 2015 | 75 | 900 | 900 | 100 | 75 (100) |
| CO | 2016 | 75 | 900 | 900 | 100 | 75 (100) |
| CO | 2017 | 75 | 900 | 900 | 100 | 75 (100) |
| CO | 2018 | 76 | 912 | 912 | 100 | 76 (100) |
| CO | 2019 | 76 | 912 | 912 | 100 | 76 (100) |
| O_3_ | 2012 | 74 | 885 | 888 | 99.66 | 72 (97.3) |
| O_3_ | 2013 | 74 | 886 | 888 | 99.77 | 73 (98.65) |
| O_3_ | 2014 | 74 | 888 | 888 | 100 | 74 (100) |
| O_3_ | 2015 | 74 | 888 | 888 | 100 | 74 (100) |
| O_3_ | 2016 | 74 | 888 | 888 | 100 | 74 (100) |
| O_3_ | 2017 | 74 | 888 | 888 | 100 | 74 (100) |
| O_3_ | 2018 | 75 | 900 | 900 | 100 | 75 (100) |
| O_3_ | 2019 | 75 | 900 | 900 | 100 | 75 (100) |

For each pollutant-year, represented stations were defined as monitoring stations with at least one available monthly observation. Expected station-months were calculated as the number of represented stations × 12. Monthly completeness was calculated as the number of available station-month observations divided by the expected number of station-month observations. Duplicate administrative-area records corresponding to the same pollutant, station, year, and month were collapsed because all duplicated records had identical concentration values.

**Table S2. Composition of COPD cases according to ascertainment criteria.**

| **COPD ascertainment** | **n** | **%** |
| --- | --- | --- |
| Spirometry-defined criterion (including overlap) | 7269 | 96.07 |
| Self-reported criterion (including overlap) | 397 | 5.25 |
| Spirometry-defined only | 7169 | 94.75 |
| Self-report only | 297 | 3.93 |
| Both criteria | 100 | 1.32 |
| Total COPD | 7566 | 100 |

**Table S3. Pearson correlations among pre-recruitment exposure-averaging periods for each air pollutant**

| **Pollutant** | **1- vs 3-month** | **1- vs 6-month** | **1- vs 12-month** | **3- vs 6-month** | **3- vs 12-month** | **6- vs 12-month** |
| --- | --- | --- | --- | --- | --- | --- |
| PM_10_ | 0.884 | 0.660 | 0.657 | 0.876 | 0.734 | 0.857 |
| PM_2.5_ | 0.873 | 0.607 | 0.592 | 0.855 | 0.672 | 0.817 |
| NO_2_ | 0.934 | 0.799 | 0.801 | 0.932 | 0.853 | 0.919 |
| NO | 0.897 | 0.822 | 0.828 | 0.978 | 0.926 | 0.948 |
| NOx | 0.924 | 0.853 | 0.853 | 0.961 | 0.908 | 0.953 |
| SO_2_ | 0.953 | 0.907 | 0.898 | 0.969 | 0.937 | 0.970 |
| CO | 0.944 | 0.843 | 0.848 | 0.948 | 0.889 | 0.937 |
| O_3_ | 0.710 | 0.478 | 0.454 | 0.767 | 0.644 | 0.869 |

Values are Pearson correlation coefficients calculated in the matched analytical sample. The 1-, 3-, 6-, and 12-month averages overlap by construction.

**Table S4. Pearson correlations among air pollutants according to pre-recruitment exposure-averaging period**

| **Pollutant** | **PM_10_** | **PM_2.5_** | **NO_2_** | NO | **NOx** | **SO_2_** | **CO** | **O_3_** |
| --- | --- | --- | --- | --- | --- | --- | --- | --- |
| **1-month exposure average** | | | | | | | | |
| PM_10_ | 1 |  |  |  |  |  |  |  |
| PM_2.5_ | 0.891 | 1 |  |  |  |  |  |  |
| NO_2_ | 0.545 | 0.535 | 1 |  |  |  |  |  |
| NO | 0.108 | 0.08 | 0.604 | 1 |  |  |  |  |
| NOx | 0.438 | 0.398 | 0.944 | 0.718 | 1 |  |  |  |
| SO_2_ | 0.506 | 0.453 | 0.549 | 0.234 | 0.486 | 1 |  |  |
| CO | 0.398 | 0.381 | 0.843 | 0.754 | 0.887 | 0.345 | 1 |  |
| O_3_ | 0.293 | 0.347 | -0.091 | -0.332 | -0.209 | -0.063 | -0.103 | 1 |
| **3-month exposure average** | | | | | | | | |
| PM_10_ | 1 |  |  |  |  |  |  |  |
| PM_2.5_ | 0.896 | 1 |  |  |  |  |  |  |
| NO_2_ | 0.534 | 0.522 | 1 |  |  |  |  |  |
| NO | 0.113 | 0.076 | 0.631 | 1 |  |  |  |  |
| NOx | 0.432 | 0.385 | 0.941 | 0.738 | 1 |  |  |  |
| SO_2_ | 0.51 | 0.45 | 0.55 | 0.234 | 0.484 | 1 |  |  |
| CO | 0.373 | 0.349 | 0.85 | 0.795 | 0.901 | 0.33 | 1 |  |
| O_3_ | 0.219 | 0.296 | -0.193 | -0.381 | -0.294 | -0.111 | -0.216 | 1 |
| **6-month exposure average** | | | | | | | | |
| PM_10_ | 1 |  |  |  |  |  |  |  |
| PM_2.5_ | 0.893 | 1 |  |  |  |  |  |  |
| NO_2_ | 0.486 | 0.462 | 1 |  |  |  |  |  |
| NO | 0.106 | 0.052 | 0.655 | 1 |  |  |  |  |
| NOx | 0.385 | 0.325 | 0.939 | 0.781 | 1 |  |  |  |
| SO_2_ | 0.561 | 0.508 | 0.596 | 0.265 | 0.518 | 1 |  |  |
| CO | 0.312 | 0.272 | 0.84 | 0.829 | 0.91 | 0.366 | 1 |  |
| O_3_ | 0.072 | 0.168 | -0.39 | -0.495 | -0.472 | -0.198 | -0.424 | 1 |
| **12-month exposure average** | | | | | | | | |
| PM_10_ | 1 |  |  |  |  |  |  |  |
| PM_2.5_ | 0.883 | 1 |  |  |  |  |  |  |
| NO_2_ | 0.401 | 0.353 | 1 |  |  |  |  |  |
| NO | 0.092 | 0.009 | 0.696 | 1 |  |  |  |  |
| NOx | 0.295 | 0.214 | 0.934 | 0.859 | 1 |  |  |  |
| SO_2_ | 0.627 | 0.595 | 0.652 | 0.285 | 0.533 | 1 |  |  |
| CO | 0.215 | 0.139 | 0.822 | 0.888 | 0.919 | 0.396 | 1 |  |
| O_3_ | 0.012 | 0.136 | -0.556 | -0.587 | -0.619 | -0.193 | -0.599 | 1 |

Values are Pearson correlation coefficients calculated in the matched analytical sample (n = 30,264). Correlations were calculated separately for the 1-, 3-, 6-, and 12-month pre-recruitment exposure averages. PM_10_, particulate matter with an aerodynamic diameter ≤ 10 µm; PM_2.5_, particulate matter with an aerodynamic diameter ≤ 2.5 µm; NO_2_, nitrogen dioxide; NO, nitric oxide (nitrogen monoxide); NOx, nitrogen oxides; SO_2_, sulfur dioxide; CO, carbon monoxide; O_3_, ozone.

**Table S5.** **Associations between air pollution exposure and COPD risk**

|  | **OR (95% CI)** | |
| --- | --- | --- |
|  | Crude model | Adjusted model^†^ |
| **PM_10_** |  |  |
| PM_10_, 1-month | **1.145 (1.131 – 1.160)*** | **1.145 (1.131 – 1.160)*** |
| PM_10_, 3-month | **1.159 (1.143 – 1.175)*** | **1.159 (1.143 – 1.175)*** |
| PM_10_, 6-month | **1.183 (1.165 – 1.201)*** | **1.183 (1.166 – 1.201)*** |
| PM_10_, 12-month | **1.298 (1.276 – 1.321)*** | **1.299 (1.277 – 1.322)*** |
| **PM_2.5_** |  |  |
| PM_2.5_, 1-month | **1.251 (1.221 – 1.282)*** | **1.252 (1.222 – 1.283)*** |
| PM_2.5_, 3-month | **1.259 (1.226 – 1.293)*** | **1.259 (1.227 – 1.293)*** |
| PM_2.5_, 6-month | **1.301 (1.264 – 1.339)*** | **1.301 (1.264 – 1.339)*** |
| PM_2.5_, 12-month | **1.641 (1.587 – 1.697)*** | **1.642 (1.588 – 1.699)*** |
| **NO_2_** |  |  |
| NO_2_, 1-month | **1.039 (1.035 – 1.044)*** | **1.039 (1.035 – 1.043)*** |
| NO_2_, 3-month | **1.038 (1.033 – 1.042)*** | **1.037 (1.033 – 1.042)*** |
| NO_2_, 6-month | **1.038 (1.033 – 1.042)*** | **1.037 (1.033 – 1.042)*** |
| NO_2_, 12-month | **1.051 (1.046 – 1.056)*** | **1.051 (1.045 – 1.056)*** |
| **NO** |  |  |
| NO, 1-month | **1.053 (1.047 – 1.059)*** | **1.053 (1.047 – 1.059)*** |
| NO, 3-month | **1.052 (1.046 – 1.058)*** | **1.052 (1.046 – 1.058)*** |
| NO, 6-month | **1.056 (1.049 – 1.062)*** | **1.055 (1.049 – 1.062)*** |
| NO, 12-month | **1.059 (1.053 – 1.066)*** | **1.059 (1.052 – 1.065)*** |
| **NOx** |  |  |
| NOx, 1-month | **1.027 (1.025 – 1.030)*** | **1.027 (1.025 – 1.030)*** |
| NOx, 3-month | **1.026 (1.023 – 1.029)*** | **1.026 (1.023 – 1.029)*** |
| NOx, 6-month | **1.026 (1.023 – 1.029)*** | **1.026 (1.023 – 1.029)*** |
| NOx, 12-month | **1.029 (1.026 – 1.032)*** | **1.029 (1.026 – 1.032)*** |
| **SO_2_** |  |  |
| SO_2_, 1-month | **1.292 (1.266 – 1.319)*** | **1.293 (1.266 – 1.319)*** |
| SO_2_, 3-month | **1.329 (1.301 – 1.358)*** | **1.330 (1.302 – 1.358)*** |
| SO_2_, 6-month | **1.374 (1.344 – 1.404)*** | **1.374 (1.344 – 1.404)*** |
| SO_2_, 12-month | **1.386 (1.356 – 1.417)*** | **1.386 (1.356 – 1.417)*** |
| **CO** |  |  |
| CO, 1-month | **3.509 (3.074 – 4.006)*** | **3.498 (3.064 – 3.994)*** |
| CO, 3-month | **3.323 (2.896 – 3.812)*** | **3.311 (2.885 – 3.799)*** |
| CO, 6-month | **3.402 (2.942 – 3.935)*** | **3.381 (2.923 – 3.911)*** |
| CO, 12-month | **4.605 (3.947 – 5.373)*** | **4.577 (3.922 – 5.342)*** |
| **O_3_** |  |  |
| O_3_, 1-month | **0.994 (0.990 – 0.998)*** | **0.994 (0.990 – 0.998)*** |
| O_3_, 3-month | **0.989 (0.984 – 0.994)*** | **0.989 (0.984 – 0.994)*** |
| O_3_, 6-month | **0.982 (0.976 – 0.989)*** | **0.982 (0.976 – 0.989)*** |
| O_3_, 12-month | 1.003 (0.996 – 1.011) | 1.003 (0.996 – 1.011) |

PM_10_, particulate matter with an aerodynamic diameter ≤ 10 µm; PM_2.5_, particulate matter with an aerodynamic diameter ≤ 2.5 µm; NO_2_, nitrogen dioxide; NO, nitric oxide (nitrogen monoxide); NOx, nitrogen oxides; SO_2_, sulfur dioxide; CO, carbon monoxide; O_3_, ozone. Odds ratios (ORs) indicate the risk based on logistic regression models, per 10 µg/m^3^ increase in PM_10_ and PM_2.5_ concentrations, and per 1-unit increase in concentrations of other pollutants

^†^adjusted for age, sex, body mass index (BMI), and smoking experience

**p* <0.05

**Table S6. Sensitivity analysis of associations between air pollution exposure and COPD with additional adjustment for smoking duration and intensity**

|  | **OR (95% CI)** | | |
| --- | --- | --- | --- |
|  | Primary model, full sample | Smoking-status model, sensitivity sample | Detailed-smoking model, sensitivity sample |
| **PM_10_** |  |  |  |
| PM_10_, 1-month | **1.145 (1.131 – 1.160)*** | **1.140 (1.125 – 1.155)*** | **1.139 (1.125 – 1.154)*** |
| PM_10_, 3-month | **1.159 (1.143 – 1.175)*** | **1.155 (1.139 – 1.172)*** | **1.155 (1.138 – 1.172)*** |
| PM_10_, 6-month | **1.183 (1.166 – 1.201)*** | **1.182 (1.163 – 1.200)*** | **1.181 (1.163 – 1.200)*** |
| PM_10_, 12-month | **1.299 (1.277 – 1.322)*** | **1.299 (1.275 – 1.322)*** | **1.298 (1.274 – 1.321)*** |
| **PM_2.5_** |  |  |  |
| PM_2.5_, 1-month | **1.252 (1.222 – 1.283)*** | **1.240 (1.209 – 1.272)*** | **1.240 (1.209 – 1.272)*** |
| PM_2.5_, 3-month | **1.259 (1.227 – 1.293)*** | **1.248 (1.215 – 1.283)*** | **1.248 (1.214 – 1.283)*** |
| PM_2.5_, 6-month | **1.301 (1.264 – 1.339)*** | **1.293 (1.255 – 1.333)*** | **1.292 (1.254 – 1.332)*** |
| PM_2.5_, 12-month | **1.642 (1.588 – 1.699)*** | **1.636 (1.580 – 1.695)*** | **1.634 (1.578 – 1.693)*** |
| **NO_2_** |  |  |  |
| NO_2_, 1-month | **1.039 (1.035 – 1.043)*** | **1.037 (1.033 – 1.042)*** | **1.037 (1.033 – 1.042)*** |
| NO_2_, 3-month | **1.037 (1.033 – 1.042)*** | **1.035 (1.031 – 1.040)*** | **1.036 (1.031 – 1.040)*** |
| NO_2_, 6-month | **1.037 (1.033 – 1.042)*** | **1.035 (1.030 – 1.040)*** | **1.036 (1.031 – 1.041)*** |
| NO_2_, 12-month | **1.051 (1.045 – 1.056)*** | **1.048 (1.042 – 1.053)*** | **1.048 (1.043 – 1.054)*** |
| **NO** |  |  |  |
| NO, 1-month | **1.053 (1.047 – 1.059)*** | **1.028 (1.023 – 1.033)*** | **1.028 (1.023 – 1.033)*** |
| NO, 3-month | **1.052 (1.046 – 1.058)*** | **1.037 (1.031 – 1.042)*** | **1.037 (1.031 – 1.042)*** |
| NO, 6-month | **1.055 (1.049 – 1.062)*** | **1.038 (1.033 – 1.044)*** | **1.038 (1.033 – 1.044)*** |
| NO, 12-month | **1.059 (1.052 – 1.065)*** | **1.046 (1.040 – 1.052)*** | **1.046 (1.040 – 1.052)*** |
| **NOx** |  |  |  |
| NOx, 1-month | **1.027 (1.025 – 1.030)*** | **1.022 (1.019 – 1.025)*** | **1.022 (1.020 – 1.025)*** |
| NOx, 3-month | **1.026 (1.023 – 1.029)*** | **1.023 (1.020 – 1.025)*** | **1.023 (1.020 – 1.025)*** |
| NOx, 6-month | **1.026 (1.023 – 1.029)*** | **1.023 (1.020 – 1.026)*** | **1.023 (1.020 – 1.026)*** |
| NOx, 12-month | **1.029 (1.026 – 1.032)*** | **1.026 (1.023 – 1.029)*** | **1.026 (1.023 – 1.029)*** |
| **SO_2_** |  |  |  |
| SO_2_, 1-month | **1.293 (1.266 – 1.319)*** | **1.270 (1.244 – 1.297)*** | **1.270 (1.244 – 1.297)*** |
| SO_2_, 3-month | **1.330 (1.302 – 1.358)*** | **1.308 (1.280 – 1.337)*** | **1.308 (1.280 – 1.337)*** |
| SO_2_, 6-month | **1.374 (1.344 – 1.404)*** | **1.347 (1.318 – 1.378)*** | **1.347 (1.317 – 1.378)*** |
| SO_2_, 12-month | **1.386 (1.356 – 1.417)*** | **1.372 (1.341 – 1.403)*** | **1.371 (1.341 – 1.403)*** |
| **CO** |  |  |  |
| CO, 1-month | **3.498 (3.064 – 3.994)*** | **3.087 (2.706 – 3.521)*** | **3.095 (2.713 – 3.530)*** |
| CO, 3-month | **3.311 (2.885 – 3.799)*** | **3.145 (2.728 – 3.625)*** | **3.159 (2.740 – 3.642)*** |
| CO, 6-month | **3.381 (2.923 – 3.911)*** | **3.255 (2.799 – 3.786)*** | **3.270 (2.811 – 3.804)*** |
| CO, 12-month | **4.577 (3.922 – 5.342)*** | **4.371 (3.724 – 5.131)*** | **4.387 (3.737 – 5.149)*** |
| **O_3_** |  |  |  |
| O_3_, 1-month | **0.994 (0.990 – 0.998)*** | **0.994 (0.990 – 0.998)*** | **0.994 (0.990 – 0.998)*** |
| O_3_, 3-month | **0.989 (0.984 – 0.994)*** | **0.990 (0.985 – 0.995)*** | **0.990 (0.985 – 0.995)*** |
| O_3_, 6-month | **0.982 (0.976 – 0.989)*** | **0.985 (0.979 – 0.992)*** | **0.985 (0.979 – 0.992)*** |
| O_3_, 12-month | 1.003 (0.996 – 1.011) | 1.007 (0.999 – 1.015) | 1.007 (0.999 – 1.015) |

PM_10_, particulate matter with an aerodynamic diameter ≤ 10 µm; PM_2.5_, particulate matter with an aerodynamic diameter ≤ 2.5 µm; NO_2_, nitrogen dioxide; NO, nitric oxide (nitrogen monoxide); NOx, nitrogen oxides; SO_2_, sulfur dioxide; CO, carbon monoxide; O_3_, ozone. Values are odds ratios (ORs) with 95% confidence intervals (CIs) from logistic regression models. The primary model was fitted in the full matched sample (n = 30,264) and adjusted for age, sex, body mass index (BMI), and smoking experience. The restricted-sample model was fitted among participants with complete detailed smoking information (n = 27,696) and adjusted for age, sex, BMI, and smoking status (never, former, or current). The detailed-smoking model additionally adjusted for smoking duration and smoking intensity. Smoking duration was expressed in years, and smoking intensity was standardized to packs per day according to the reported daily, weekly, or monthly smoking frequency. PM_10_ and PM_2.5_ estimates are expressed per 10 µg/m^3^ increase; other pollutants are expressed per 1-unit increase.

**p* <0.05

**Table S7. Associations between air pollution exposure and COPD restricted to spirometry-defined airflow obstruction**

|  | **OR (95% CI) ^†^** |
| --- | --- |
| **PM_10_** |  |
| PM_10_, 1-month | **1.151 (1.136 – 1.165)*** |
| PM_10_, 3-month | **1.164 (1.148 – 1.181)*** |
| PM_10_, 6-month | **1.190 (1.172 – 1.208)*** |
| PM_10_, 12-month | **1.312 (1.289 – 1.335)*** |
| **PM_2.5_** |  |
| PM_2.5_, 1-month | **1.262 (1.231 – 1.294)*** |
| PM_2.5_, 3-month | **1.268 (1.235 – 1.303)*** |
| PM_2.5_, 6-month | **1.312 (1.274 – 1.351)*** |
| PM_2.5_, 12-month | **1.671 (1.614 – 1.729)*** |
| **NO_2_** |  |
| NO_2_, 1-month | **1.041 (1.037 – 1.046)*** |
| NO_2_, 3-month | **1.039 (1.035 – 1.044)*** |
| NO_2_, 6-month | **1.039 (1.034 – 1.044)*** |
| NO_2_, 12-month | **1.053 (1.048 – 1.059)*** |
| **NO** |  |
| NO, 1-month | **1.054 (1.048 – 1.060)*** |
| NO, 3-month | **1.053 (1.047 – 1.060)*** |
| NO, 6-month | **1.057 (1.050 – 1.063)*** |
| NO, 12-month | **1.060 (1.054 – 1.067)*** |
| **NOx** |  |
| NOx, 1-month | **1.028 (1.026 – 1.031)*** |
| NOx, 3-month | **1.027 (1.024 – 1.030)*** |
| NOx, 6-month | **1.027 (1.024 – 1.030)*** |
| NOx, 12-month | **1.030 (1.027 – 1.033)*** |
| **SO_2_** |  |
| SO_2_, 1-month | **1.306 (1.279 – 1.333)*** |
| SO_2_, 3-month | **1.344 (1.315 – 1.373)*** |
| SO_2_, 6-month | **1.390 (1.359 – 1.421)*** |
| SO_2_, 12-month | **1.403 (1.372–1.435)*** |
| **CO** |  |
| CO, 1-month | **3.658 (3.199 – 4.183)*** |
| CO, 3-month | **3.450 (3.001 – 3.965)*** |
| CO, 6-month | **3.519 (3.036 – 4.078)*** |
| CO, 12-month | **4.786 (4.094 – 5.595)*** |
| **O_3_** |  |
| O_3_, 1-month | **0.994 (0.990 – 0.997)*** |
| O_3_, 3-month | **0.989 (0.984 – 0.994)*** |
| O_3_, 6-month | **0.982 (0.975 – 0.988)*** |
| O_3_, 12-month | 1.003 (0.995 – 1.011) |

PM_10_, particulate matter with an aerodynamic diameter ≤ 10 µm; PM_2.5_, particulate matter with an aerodynamic diameter ≤ 2.5 µm; NO_2_, nitrogen dioxide; NO, nitric oxide (nitrogen monoxide); NOx, nitrogen oxides; SO_2_, sulfur dioxide; CO, carbon monoxide; O_3_, ozone. The sensitivity analysis included 7,269 participants with spirometry-defined airflow obstruction and 22,698 controls. Odds ratios (ORs) indicate the risk based on logistic regression models, per 10 µg/m^3^ increase in PM_10_ and PM_2.5_ concentrations, and per 1-unit increase in concentrations of other pollutants

^†^adjusted for age, sex, body mass index (BMI), and smoking experience

**p* <0.05

**Table S8. Associations between air pollution exposure and COPD according to mutually exclusive ascertainment subgroups**

|  | **OR (95% CI)^†^** | | |
| --- | --- | --- | --- |
|  | Spirometry-defined only  (n = 7,169) | Self-report only  (n = 297) | Both criteria  (n = 100) |
| **PM_10_** |  |  |  |
| PM_10_, 1-month | **1.151 (1.137 – 1.166)*** | 1.016 (0.957 – 1.079) | 1.075 (0.977 – 1.184) |
| PM_10_, 3-month | **1.165 (1.148 – 1.181)*** | 1.030 (0.966 – 1.098) | **1.137 (1.030 – 1.256)*** |
| PM_10_, 6-month | **1.190 (1.172 – 1.208)*** | 1.019 (0.951 – 1.093) | **1.198 (1.077 – 1.332)*** |
| PM_10_, 12-month | **1.312 (1.289 – 1.335)** | 1.022 (0.946 – 1.105) | **1.308 (1.153 – 1.484)*** |
| **PM_2.5_** |  |  |  |
| PM_2.5_, 1-month | **1.263 (1.232 – 1.295)*** | 1.011 (0.901 – 1.135) | 1.151 (0.958 – 1.383) |
| PM_2.5_, 3-month | **1.268 (1.235 – 1.303)*** | 1.050 (0.929 – 1.187) | **1.266 (1.046 – 1.532)*** |
| PM_2.5_, 6-month | **1.311 (1.273 – 1.350)*** | 1.049 (0.919 – 1.197) | **1.379 (1.126 – 1.689)*** |
| PM_2.5_, 12-month | **1.669 (1.613 – 1.728)*** | 1.082 (0.932 – 1.256) | **1.684 (1.332 – 2.128)*** |
| **NO_2_** |  |  |  |
| NO_2_, 1-month | **1.041 (1.037 – 1.046)*** | 0.988 (0.969 – 1.007) | 1.019 (0.988 – 1.051) |
| NO_2_, 3-month | **1.039 (1.035 – 1.044)*** | 0.994 (0.974 – 1.014) | 1.024 (0.991 – 1.058) |
| NO_2_, 6-month | **1.039 (1.034 – 1.044)*** | 0.994 (0.974 – 1.015) | 1.027 (0.992 – 1.063) |
| NO_2_, 12-month | **1.054 (1.048 – 1.059)*** | 0.994 (0.972 – 1.016) | 1.030 (0.991 – 1.069) |
| **NO** |  |  |  |
| NO, 1-month | **1.055 (1.049 – 1.061)*** | 1.019 (0.993 – 1.045) | 1.020 (0.976 – 1.065) |
| NO, 3-month | **1.054 (1.047 – 1.060)*** | 1.015 (0.988 – 1.043) | 1.013 (0.967 – 1.061) |
| NO, 6-month | **1.057 (1.051 – 1.064)*** | 1.019 (0.992 – 1.048) | 1.012 (0.963 – 1.063) |
| NO, 12-month | **1.061 (1.054 – 1.067)*** | 1.020 (0.992 – 1.049) | 1.012 (0.962 – 1.064) |
| **NOx** |  |  |  |
| NOx, 1-month | **1.029 (1.026 – 1.031)*** | 1.000 (0.987 – 1.012) | 1.011 (0.991 – 1.031) |
| NOx, 3-month | **1.027 (1.024 – 1.030)*** | 1.004 (0.991 – 1.016) | 1.012 (0.992 – 1.033) |
| NOx, 6-month | **1.027 (1.024 – 1.030)*** | 1.003 (0.990 – 1.016) | 1.012 (0.991 – 1.033) |
| NOx, 12-month | **1.030 (1.027 – 1.033)*** | 1.002 (0.988 – 1.015) | 1.011 (0.989 – 1.033) |
| **SO_2_** |  |  |  |
| SO_2_, 1-month | **1.307 (1.280 – 1.335)*** | 0.957 (0.865 – 1.059) | **1.160 (1.004 – 1.341)*** |
| SO_2_, 3-month | **1.344 (1.316 – 1.374)*** | 0.977 (0.882 – 1.083) | **1.226 (1.064 – 1.413)*** |
| SO_2_, 6-month | **1.390 (1.359 – 1.421)*** | 0.989 (0.891 – 1.098) | **1.290 (1.117 – 1.488)*** |
| SO_2_, 12-month | **1.403 (1.372 – 1.435)*** | 0.992 (0.894 – 1.102) | **1.321 (1.146 – 1.523)*** |
| **CO** |  |  |  |
| CO, 1-month | **3.696 (3.230 – 4.228)*** | 1.050 (0.559 – 1.971) | 1.367 (0.486 – 3.841) |
| CO, 3-month | **3.478 (3.025 – 4.000)*** | 1.126 (0.588 – 2.157) | 1.550 (0.537 – 4.468) |
| CO, 6-month | **3.544 (3.057 – 4.109)*** | 1.178 (0.592 – 2.342) | 1.675 (0.549 – 5.105) |
| CO, 12-month | **4.829 (4.129 – 5.648)*** | 1.344 (0.652 – 2.771) | 1.721 (0.523 – 5.659) |
| **O_3_** |  |  |  |
| O_3_, 1-month | **0.994 (0.990 – 0.998)*** | 0.999 (0.982 – 1.016) | 0.992 (0.964 – 1.022) |
| O_3_, 3-month | **0.988 (0.983 – 0.994)*** | 1.008 (0.986 – 1.030) | 1.002 (0.965 – 1.041) |
| O_3_, 6-month | **0.981 (0.974 – 0.988)*** | 1.002 (0.974 – 1.032) | 1.028 (0.980 – 1.080) |
| O_3_, 12-month | 1.003 (0.996 – 1.011) | 1.007 (0.972 – 1.043) | 1.051 (0.992 – 1.114) |

PM_10_, particulate matter with an aerodynamic diameter ≤ 10 µm; PM_2.5_, particulate matter with an aerodynamic diameter ≤ 2.5 µm; NO_2_, nitrogen dioxide; NO, nitric oxide (nitrogen monoxide); NOx, nitrogen oxides; SO_2_, sulfur dioxide; CO, carbon monoxide; O_3_, ozone. Odds ratios (ORs) indicate the risk based on logistic regression models, per 10 µg/m^3^ increase in PM_10_ and PM_2.5_ concentrations, and per 1-unit increase in concentrations of other pollutants

^†^adjusted for age, sex, body mass index (BMI), and smoking experience

**p* <0.05

**Table S9. Associations between air pollution exposure and COPD status among participants with anemia (COPD with anemia vs. controls with anemia)**

|  | **OR (95% CI)** | |
| --- | --- | --- |
|  | Crude model | Adjusted model^†^ |
| **PM_10_** |  |  |
| PM_10_, 1-month | **1.151 (1.108 – 1.196)*** | **1.150 (1.107 – 1.196)*** |
| PM_10_, 3-month | **1.170 (1.122 – 1.220)*** | **1.169 (1.121 – 1.220)*** |
| PM_10_, 6-month | **1.204 (1.152 – 1.259)*** | **1.204 (1.151 – 1.259)*** |
| PM_10_, 12-month | **1.336 (1.268 – 1.406)*** | **1.337 (1.269 – 1.409)*** |
| **PM_2.5_** |  |  |
| PM_2.5_, 1-month | **1.227 (1.140 – 1.321)*** | **1.225 (1.137 – 1.319)*** |
| PM_2.5_, 3-month | **1.246 (1.149 – 1.351)*** | **1.243 (1.145 – 1.348)*** |
| PM_2.5_, 6-month | **1.303 (1.196 – 1.420)*** | **1.300 (1.192 – 1.418)*** |
| PM_2.5_, 12-month | **1.678 (1.517 – 1.857)*** | **1.679 (1.517 – 1.859)*** |
| **NO_2_** |  |  |
| NO_2_, 1-month | **1.038 (1.025 – 1.051)*** | **1.037 (1.024 – 1.050)*** |
| NO_2_, 3-month | **1.037 (1.023 – 1.050)*** | **1.036 (1.023 – 1.050)*** |
| NO_2_, 6-month | **1.039 (1.025 – 1.053)*** | **1.038 (1.024 – 1.053)*** |
| NO_2_, 12-month | **1.052 (1.036 – 1.068)*** | **1.051 (1.036 – 1.067)*** |
| **NO** |  |  |
| NO, 1-month | **1.050 (1.032 – 1.069)*** | **1.051 (1.033 – 1.070)*** |
| NO, 3-month | **1.052 (1.033 – 1.070)*** | **1.052 (1.034 – 1.071)*** |
| NO, 6-month | **1.058 (1.039 – 1.078)*** | **1.059 (1.040 – 1.078)*** |
| NO, 12-month | **1.064 (1.045 – 1.083)*** | **1.064 (1.045 – 1.084)*** |
| **NOx** |  |  |
| NOx, 1-month | **1.027 (1.019 – 1.035)*** | **1.027 (1.019 – 1.035)*** |
| NOx, 3-month | **1.026 (1.018 – 1.034)*** | **1.026 (1.017 – 1.034)*** |
| NOx, 6-month | **1.027 (1.019 – 1.035)*** | **1.027 (1.018 – 1.035)*** |
| NOx, 12-month | **1.030 (1.022 – 1.039)*** | **1.030 (1.021 – 1.039)*** |
| **SO_2_** |  |  |
| SO_2_, 1-month | **1.266 (1.194 – 1.343)*** | **1.267 (1.194 – 1.344)*** |
| SO_2_, 3-month | **1.299 (1.223 – 1.379)*** | **1.300 (1.224 – 1.381)*** |
| SO_2_, 6-month | **1.357 (1.275 – 1.445)*** | **1.359 (1.276 – 1.447)*** |
| SO_2_, 12-month | **1.377 (1.293 – 1.467)*** | **1.379 (1.294 – 1.469)*** |
| **CO** |  |  |
| CO, 1-month | **3.693 (2.506 – 5.440)*** | **3.686 (2.499 – 5.436)*** |
| CO, 3-month | **3.537 (2.363 – 5.294)*** | **3.536 (2.360 – 5.299)*** |
| CO, 6-month | **4.129 (2.694 – 6.326)*** | **4.130 (2.691 – 6.337)*** |
| CO, 12-month | **5.629 (3.572 – 8.871)*** | **5.655 (3.583 – 8.927)*** |
| **O_3_** |  |  |
| O_3_, 1-month | 0.991 (0.981 – 1.002) | 0.991 (0.980 – 1.002) |
| O_3_, 3-month | **0.981 (0.966 – 0.996)*** | **0.981 (0.966 – 0.996)*** |
| O_3_, 6-month | **0.970 (0.950 – 0.989)*** | **0.969 (0.949 – 0.989)*** |
| O_3_, 12-month | 0.988 (0.965 – 1.012) | 0.987 (0.965 – 1.011) |

PM_10_, particulate matter with an aerodynamic diameter ≤ 10 µm; PM_2.5_, particulate matter with an aerodynamic diameter ≤ 2.5 µm; NO_2_, nitrogen dioxide; NO, nitric oxide (nitrogen monoxide); NOx, nitrogen oxides; SO_2_, sulfur dioxide; CO, carbon monoxide; O_3_, ozone. Odds ratios (ORs) indicate the risk based on logistic regression models, per 10 µg/m^3^ increase in PM_10_ and PM_2.5_ concentrations, and per 1-unit increase in concentrations of other pollutants

^†^adjusted for age, sex, body mass index (BMI), and smoking experience

**p* <0.05

**Table S10. Overall anemia analysis: Associations between air pollution exposure and anemia status in the full matched analytic sample**

|  | **OR (95% CI)** | |
| --- | --- | --- |
|  | Crude model | Adjusted model^†^ |
| **PM_10_** |  |  |
| 1-month | **0.979 (0.961 – 0.997)*** | **0.974 (0.956 – 0.993)*** |
| 3-month | **0.975 (0.956 – 0.995)*** | **0.970 (0.950 – 0.990)*** |
| 6-month | 0.988 (0.968 – 1.009) | **0.978 (0.957 – 0.999)*** |
| 12-month | 1.001 (0.978 – 1.024) | 0.985 (0.961 – 1.008) |
| **PM_2.5_** |  |  |
| 1-month | **0.954 (0.921 – 0.988)*** | **0.944 (0.911 – 0.979)*** |
| 3-month | **0.946 (0.911 – 0.982)*** | **0.935 (0.900 – 0.972)*** |
| 6-month | 0.973 (0.934 – 1.013) | **0.952 (0.914 – 0.993)*** |
| 12-month | 1.002 (0.958 – 1.049) | 0.971 (0.926 – 1.017) |
| **NO_2_** |  |  |
| 1-month | **0.994 (0.988 – 1.000)*** | 0.994 (0.988 – 1.000) |
| 3-month | 0.994 (0.988 – 1.000) | 0.995 (0.988 – 1.001) |
| 6-month | 0.997 (0.991 – 1.004) | 0.997 (0.991 – 1.004) |
| 12-month | 1.000 (0.993 – 1.007) | 0.999 (0.992 – 1.006) |
| **NO** |  |  |
| 1-month | 0.996 (0.987 – 1.005) | 0.996 (0.987 – 1.005) |
| 3-month | 0.997 (0.988 – 1.006) | 0.997 (0.988 – 1.006) |
| 6-month | 0.999 (0.990 – 1.008) | 0.999 (0.990 – 1.008) |
| 12-month | 1.000 (0.991 – 1.009) | 1.000 (0.991 – 1.010) |
| **NOx** |  |  |
| 1-month | 0.997 (0.993 – 1.000) | 0.997 (0.993 – 1.001) |
| 3-month | 0.997 (0.993 – 1.001) | 0.997 (0.993 – 1.001) |
| 6-month | 0.999 (0.995 – 1.003) | 0.999 (0.995 – 1.003) |
| 12-month | 1.000 (0.996 – 1.004) | 1.000 (0.995 – 1.004) |
| **SO_2_** |  |  |
| 1-month | 1.008 (0.979 – 1.037) | 0.998 (0.968 – 1.028) |
| 3-month | 1.010 (0.981 – 1.041) | 1.001 (0.970 – 1.032) |
| 6-month | 1.014 (0.984 – 1.046) | 1.003 (0.972 – 1.035) |
| 12-month | 1.022 (0.991 – 1.054) | 1.010 (0.979 – 1.042) |
| **CO** |  |  |
| 1-month | 0.859 (0.708 – 1.043) | 0.879 (0.720 – 1.072) |
| 3-month | 0.870 (0.711 – 1.064) | 0.894 (0.727 – 1.098) |
| 6-month | 0.947 (0.767 – 1.170) | 0.958 (0.771 – 1.189) |
| 12-month | 1.011 (0.807 – 1.266) | 1.011 (0.802 – 1.274) |
| **O_3_** |  |  |
| 1-month | 0.995 (0.990 – 1.000) | 0.996 (0.991 – 1.001) |
| 3-month | **0.992 (0.985 – 0.999)*** | **0.993 (0.986 – 1.000)*** |
| 6-month | **0.990 (0.981 – 0.999)*** | **0.990 (0.981 – 0.999)*** |
| 12-month | 0.994 (0.983 – 1.004) | 0.991 (0.981 – 1.002) |

PM_10_, particulate matter with an aerodynamic diameter ≤ 10 µm; PM_2.5_, particulate matter with an aerodynamic diameter ≤ 2.5 µm; NO_2_, nitrogen dioxide; NO, nitric oxide (nitrogen monoxide); NOx, nitrogen oxides; SO_2_, sulfur dioxide; CO, carbon monoxide; O_3_, ozone. Odds ratios (ORs) indicate the risk based on logistic regression models, per 10 µg/m^3^ increase in PM_10_ and PM_2.5_ concentrations, and per 1-unit increase in concentrations of other pollutants. Anemia was modeled as the binary outcome regardless of COPD status, with participants without anemia serving as the reference group.

^†^adjusted for age, sex, body mass index (BMI), and smoking experience

**p* <0.05

**Table S11. Within-COPD anemia analysis: Associations between air pollution exposure and anemia status among participants with COPD**

|  | **OR (95% CI)** | |
| --- | --- | --- |
|  | Crude model | Adjusted model^†^ |
| **PM_10_** |  |  |
| 1-month | 0.978 (0.945 – 1.012) | 0.981 (0.947 – 1.017) |
| 3-month | 0.974 (0.938 – 1.011) | 0.976 (0.939 – 1.014) |
| 6-month | 0.997 (0.957 – 1.038) | 0.992 (0.952 – 1.034) |
| 12-month | 1.014 (0.968 – 1.061) | 1.003 (0.957 – 1.051) |
| **PM_2.5_** |  |  |
| 1-month | **0.934 (0.875 – 0.997)*** | 0.938 (0.878 – 1.003) |
| 3-month | **0.925 (0.861 – 0.994)*** | **0.929 (0.863 – 1.000)*** |
| 6-month | 0.963 (0.890 – 1.042) | 0.955 (0.881 – 1.036) |
| 12-month | 0.996 (0.909 – 1.091) | 0.976 (0.888 – 1.072) |
| **NO_2_** |  |  |
| 1-month | 0.991 (0.980 – 1.002) | 0.991 (0.980 – 1.002) |
| 3-month | 0.992 (0.980 – 1.003) | 0.991 (0.980 – 1.003) |
| 6-month | 0.997 (0.985 – 1.009) | 0.995 (0.983 – 1.008) |
| 12-month | 0.999 (0.986 – 1.013) | 0.997 (0.983 – 1.011) |
| **NO** |  |  |
| 1-month | 0.989 (0.973 – 1.006) | 0.989 (0.972 – 1.005) |
| 3-month | 0.994 (0.977 – 1.010) | 0.993 (0.977 – 1.010) |
| 6-month | 0.999 (0.982 – 1.015) | 0.997 (0.981 – 1.014) |
| 12-month | 1.003 (0.987 – 1.019) | 1.001 (0.985 – 1.018) |
| **NOx** |  |  |
| 1-month | 0.995 (0.988 – 1.002) | 0.995 (0.988 – 1.002) |
| 3-month | 0.996 (0.988 – 1.003) | 0.996 (0.988 – 1.003) |
| 6-month | 0.999 (0.991 – 1.006) | 0.998 (0.991 – 1.006) |
| 12-month | 1.000 (0.993 – 1.008) | 0.999 (0.991 – 1.007) |
| **SO_2_** |  |  |
| 1-month | 0.993 (0.939 – 1.051) | 0.984 (0.929 – 1.042) |
| 3-month | 1.000 (0.944 – 1.060) | 0.989 (0.932 – 1.049) |
| 6-month | 1.013 (0.955 – 1.074) | 0.997 (0.939 – 1.059) |
| 12-month | 1.022 (0.964 – 1.084) | 1.004 (0.945 – 1.066) |
| **CO** |  |  |
| 1-month | 0.890 (0.620 – 1.279) | 0.886 (0.613 – 1.282) |
| 3-month | 0.912 (0.630 – 1.322) | 0.908 (0.622 – 1.326) |
| 6-month | 1.090 (0.743 – 1.599) | 1.043 (0.704 – 1.546) |
| 12-month | 1.153 (0.766 – 1.735) | 1.074 (0.705 – 1.637) |
| **O_3_** |  |  |
| 1-month | 0.994 (0.984 – 1.003) | 0.995 (0.985 – 1.005) |
| 3-month | **0.986 (0.973 – 1.000)*** | 0.989 (0.975 – 1.002) |
| 6-month | **0.980 (0.962 – 0.998)*** | 0.982 (0.963 – 1.000) |
| 12-month | 0.983 (0.963 – 1.003) | 0.982 (0.962 – 1.003) |

PM_10_, particulate matter with an aerodynamic diameter ≤ 10 µm; PM_2.5_, particulate matter with an aerodynamic diameter ≤ 2.5 µm; NO_2_, nitrogen dioxide; NO, nitric oxide (nitrogen monoxide); NOx, nitrogen oxides; SO_2_, sulfur dioxide; CO, carbon monoxide; O_3_, ozone. Odds ratios (ORs) indicate the risk based on logistic regression models, per 10 µg/m^3^ increase in PM_10_ and PM_2.5_ concentrations, and per 1-unit increase in concentrations of other pollutants

^†^adjusted for age, sex, body mass index (BMI), and smoking experience

**p* <0.05

**Table S12. COPD PRS-air pollution interaction analyses for COPD risk**

|  | **Interaction OR (95% CI)^†^** | **p-value** |
| --- | --- | --- |
| **PM_10_** |  |  |
| 1-month | 0.995 (0.983 – 1.008) | 0.434 |
| 3-month | 0.997 (0.983 – 1.010) | 0.628 |
| 6-month | 0.999 (0.984 – 1.014) | 0.891 |
| 12-month | 1.003 (0.986 – 1.021) | 0.737 |
| **PM_2.5_** |  |  |
| 1-month | 0.988 (0.965 – 1.012) | 0.334 |
| 3-month | 0.994 (0.968 – 1.020) | 0.635 |
| 6-month | 0.998 (0.969 – 1.027) | 0.868 |
| 12-month | 0.999 (0.966 – 1.034) | 0.968 |
| **NO_2_** |  |  |
| 1-month | 0.999 (0.995 – 1.003) | 0.747 |
| 3-month | 1.000 (0.995 – 1.004) | 0.856 |
| 6-month | 1.000 (0.996 – 1.005) | 0.854 |
| 12-month | 1.001 (0.996 – 1.006) | 0.632 |
| **NO** |  |  |
| 1-month | 1.000 (0.996 – 1.005) | 0.924 |
| 3-month | 1.002 (0.997 – 1.007) | 0.487 |
| 6-month | 1.002 (0.997 – 1.007) | 0.427 |
| 12-month | 1.002 (0.997 – 1.008) | 0.426 |
| **NOx** |  |  |
| 1-month | 1.000 (0.998 – 1.002) | 0.977 |
| 3-month | 1.000 (0.998 – 1.003) | 0.718 |
| 6-month | 1.001 (0.998 – 1.003) | 0.585 |
| 12-month | 1.001 (0.998 – 1.004) | 0.539 |
| **SO_2_** |  |  |
| 1-month | 1.001 (0.981 – 1.021) | 0.926 |
| 3-month | 0.999 (0.978 – 1.020) | 0.914 |
| 6-month | 0.996 (0.975 – 1.018) | 0.714 |
| 12-month | 1.000 (0.978 – 1.022) | 0.970 |
| **CO** |  |  |
| 1-month | 1.030 (0.909 – 1.168) | 0.641 |
| 3-month | 1.018 (0.889 – 1.166) | 0.798 |
| 6-month | 1.031 (0.892 – 1.192) | 0.677 |
| 12-month | 1.064 (0.912 – 1.241) | 0.428 |
| **O_3_** |  |  |
| 1-month | 0.998 (0.995 – 1.002) | 0.384 |
| 3-month | 0.999 (0.994 – 1.004) | 0.683 |
| 6-month | 0.998 (0.991 – 1.004) | 0.516 |
| 12-month | 0.998 (0.991 – 1.006) | 0.699 |

PM_10_, particulate matter with an aerodynamic diameter ≤ 10 µm; PM_2.5_, particulate matter with an aerodynamic diameter ≤ 2.5 µm; NO_2_, nitrogen dioxide; NO, nitric oxide (nitrogen monoxide); NOx, nitrogen oxides; SO_2_, sulfur dioxide; CO, carbon monoxide; O_3_, ozone. Odds ratios (ORs) indicate the risk based on logistic regression models, per 10 µg/m^3^ increase in PM_10_ and PM_2.5_ concentrations, and per 1-unit increase in concentrations of other pollutants

^†^adjusted for age, sex, body mass index (BMI), smoking experience, and principal components (PC1-PC10)

**p* <0.05

**Table S13. RBC, Hematocrit, MCV, MCH, and MCHC PRS-air pollution interaction analyses for anemia risk and corresponding indices in COPD**

|  | **Interaction OR (95% CI)^†^** | **p-value** | **Interaction β (95% CI)^†^** | **p-value** |
| --- | --- | --- | --- | --- |
|  | Anemia risk in COPD | | Corresponding indices in COPD | |
| **RBC** | | | | |
| **PM_10_** |  |  |  |  |
| 1-month | 1.025 (0.990 – 1.061) | 0.165 | -0.001 (-0.006 – 0.003) | 0.555 |
| 3-month | 1.016 (0.977 – 1.057) | 0.415 | -0.001 (-0.006 – 0.005) | 0.799 |
| 6-month | 1.016 (0.974 – 1.060) | 0.470 | -0.002 (-0.007 – 0.004) | 0.592 |
| 12-month | 1.033 (0.985 – 1.084) | 0.185 | 0.000 (-0.007 – 0.006) | 0.902 |
| **PM_2.5_** |  |  |  |  |
| 1-month | 1.045 (0.979 – 1.115) | 0.187 | -0.001 (-0.010 – 0.008) | 0.776 |
| 3-month | 1.030 (0.957 – 1.108) | 0.433 | 0.000 (-0.010 – 0.010) | 0.941 |
| 6-month | 1.029 (0.949 – 1.116) | 0.491 | -0.001 (-0.013 – 0.010) | 0.804 |
| 12-month | 1.081 (0.983 – 1.189) | 0.108 | -0.001 (-0.014 – 0.012) | 0.839 |
| **NO_2_** |  |  |  |  |
| 1-month | 1.004 (0.993 – 1.015) | 0.524 | -0.001 (-0.002 – 0.001) | 0.221 |
| 3-month | 1.004 (0.993 – 1.016) | 0.460 | -0.001 (-0.002 – 0.001) | 0.315 |
| 6-month | 1.005 (0.992 – 1.018) | 0.444 | -0.001 (-0.003 – 0.001) | 0.322 |
| 12-month | 1.010 (0.996 – 1.024) | 0.164 | -0.001 (-0.003 – 0.001) | 0.404 |
| **NO** |  |  |  |  |
| 1-month | 1.006 (0.993 – 1.020) | 0.369 | 0.001 (-0.001 – 0.003) | 0.292 |
| 3-month | 0.999 (0.986 – 1.013) | 0.941 | 0.000 (-0.002 – 0.002) | 0.850 |
| 6-month | 0.999 (0.987 – 1.012) | 0.904 | 0.000 (-0.002 – 0.002) | 0.984 |
| 12-month | 1.002 (0.988 – 1.015) | 0.821 | 0.000 (-0.002 – 0.002) | 0.816 |
| **NOx** |  |  |  |  |
| 1-month | 0.999 (0.993 – 1.005) | 0.753 | -0.001 (-0.001 – 0.000) | 0.185 |
| 3-month | 1.000 (0.993 – 1.007) | 0.917 | -0.001 (-0.002 – 0.000) | 0.257 |
| 6-month | 1.000 (0.993 – 1.008) | 0.914 | 0.000 (-0.001 – 0.001) | 0.367 |
| 12-month | 1.003 (0.995 – 1.011) | 0.446 | 0.000 (-0.001 – 0.001) | 0.500 |
| **SO_2_** |  |  |  |  |
| 1-month | 1.047 (0.990 – 1.107) | 0.105 | -0.003 (-0.011 – 0.005) | 0.469 |
| 3-month | 1.044 (0.985 – 1.107) | 0.145 | -0.003 (-0.011 – 0.006) | 0.554 |
| 6-month | 1.050 (0.989 – 1.114) | 0.107 | -0.003 (-0.011 – 0.005) | 0.480 |
| 12-month | **1.065 (1.005 – 1.128)*** | **0.034** | -0.003 (-0.012 – 0.005) | 0.440 |
| **CO** |  |  |  |  |
| 1-month | 1.035 (0.741 – 1.445) | 0.842 | -0.021 (-0.068 – 0.025) | 0.364 |
| 3-month | 1.044 (0.724 – 1.506) | 0.817 | -0.016 (-0.067 – 0.034) | 0.527 |
| 6-month | 1.014 (0.690 – 1.488) | 0.945 | -0.017 (-0.071 – 0.037) | 0.538 |
| 12-month | 1.122 (0.741 – 1.700) | 0.586 | -0.018 (-0.076 – 0.041) | 0.554 |
| **O_3_** |  |  |  |  |
| 1-month | 1.002 (0.992 – 1.012) | 0.642 | 0.000 (-0.001 – 0.001) | 0.875 |
| 3-month | 1.001 (0.987 – 1.015) | 0.894 | 0.001 (-0.001 – 0.003) | 0.227 |
| 6-month | 0.992 (0.973 – 1.010) | 0.368 | 0.001 (-0.002 – 0.004) | 0.430 |
| 12-month | 0.987 (0.967 – 1.007) | 0.199 | 0.001 (-0.002 – 0.004) | 0.376 |
| **Hematocrit** | | | | |
| **PM_10_** |  |  |  |  |
| 1-month | 1.018 (0.983 – 1.054) | 0.316 | -0.002 (-0.040 – 0.037) | 0.933 |
| 3-month | 1.023 (0.985 – 1.064) | 0.240 | -0.003 (-0.044 – 0.039) | 0.900 |
| 6-month | 1.030 (0.989 – 1.074) | 0.154 | -0.018 (-0.063 – 0.027) | 0.439 |
| 12-month | 1.032 (0.984 – 1.082) | 0.194 | -0.012 (-0.063 – 0.039) | 0.648 |
| **PM_2.5_** |  |  |  |  |
| 1-month | 1.027 (0.962 – 1.095) | 0.430 | 0.015 (-0.056 – 0.086) | 0.682 |
| 3-month | 1.043 (0.970 – 1.122) | 0.255 | 0.019 (-0.060 – 0.097) | 0.644 |
| 6-month | 1.068 (0.985 – 1.156) | 0.110 | -0.015 (-0.102 – 0.071) | 0.726 |
| 12-month | 1.097 (0.998 – 1.205) | 0.055 | -0.030 (-0.130 – 0.071) | 0.562 |
| **NO_2_** |  |  |  |  |
| 1-month | 0.999 (0.988 – 1.010) | 0.868 | 0.004 (-0.008 – 0.016) | 0.530 |
| 3-month | 1.003 (0.991 – 1.015) | 0.631 | 0.001 (-0.012 – 0.014) | 0.894 |
| 6-month | 1.005 (0.993 – 1.018) | 0.411 | -0.002 (-0.016 – 0.012) | 0.763 |
| 12-month | 1.007 (0.993 – 1.020) | 0.328 | -0.001 (-0.016 – 0.013) | 0.859 |
| **NO** |  |  |  |  |
| 1-month | 1.005 (0.993 – 1.018) | 0.420 | 0.001 (-0.013 – 0.014) | 0.933 |
| 3-month | 0.993 (0.978 – 1.008) | 0.326 | 0.008 (-0.007 – 0.023) | 0.315 |
| 6-month | 0.991 (0.976 – 1.006) | 0.219 | 0.008 (-0.007 – 0.023) | 0.308 |
| 12-month | 0.996 (0.981 – 1.011) | 0.590 | 0.004 (-0.012 – 0.020) | 0.611 |
| **NOx** |  |  |  |  |
| 1-month | 0.997 (0.990 – 1.004) | 0.362 | 0.003 (-0.004 – 0.010) | 0.441 |
| 3-month | 1.000 (0.993 – 1.006) | 0.921 | 0.001 (-0.006 – 0.008) | 0.814 |
| 6-month | 1.001 (0.994 – 1.008) | 0.830 | -0.001 (-0.008 – 0.007) | 0.876 |
| 12-month | 1.002 (0.994 – 1.009) | 0.669 | -0.001 (-0.009 – 0.008) | 0.889 |
| **SO_2_** |  |  |  |  |
| 1-month | **1.061 (1.004 – 1.121)*** | **0.036** | -0.029 (-0.091 – 0.033) | 0.365 |
| 3-month | **1.077 (1.016 – 1.141)*** | **0.012** | -0.051 (-0.115 – 0.014) | 0.121 |
| 6-month | **1.083 (1.022 – 1.149)*** | **0.008** | -0.063 (-0.129 – 0.002) | 0.059 |
| 12-month | **1.085 (1.024 – 1.149)*** | **0.006** | -0.053 (-0.118 – 0.012) | 0.108 |
| **CO** |  |  |  |  |
| 1-month | 0.944 (0.671 – 1.329) | 0.742 | 0.158 (-0.217 – 0.533) | 0.408 |
| 3-month | 0.965 (0.668 – 1.394) | 0.851 | 0.120 (-0.285 – 0.525) | 0.561 |
| 6-month | 0.957 (0.653 – 1.402) | 0.822 | 0.068 (-0.359 – 0.496) | 0.755 |
| 12-month | 0.996 (0.660 – 1.503) | 0.984 | 0.080 (-0.383 – 0.542) | 0.735 |
| **O_3_** |  |  |  |  |
| 1-month | 1.000 (0.990 – 1.010) | 0.967 | 0.001 (-0.010 – 0.011) | 0.915 |
| 3-month | 1.003 (0.990 – 1.017) | 0.622 | 0.002 (-0.013 – 0.016) | 0.826 |
| 6-month | 0.994 (0.976 – 1.012) | 0.524 | 0.004 (-0.016 – 0.024) | 0.724 |
| 12-month | 0.993 (0.973 – 1.013) | 0.488 | 0.000 (-0.022 – 0.022) | 0.991 |
| **MCV** | | | | |
| **PM_10_** |  |  |  |  |
| 1-month | 1.008 (0.973 – 1.043) | 0.665 | -0.073 (-0.157 – 0.011) | 0.087 |
| 3-month | 1.031 (0.993 – 1.071) | 0.113 | -0.061 (-0.154 – 0.031) | 0.196 |
| 6-month | 1.041 (0.999 – 1.085) | 0.056 | -0.057 (-0.160 – 0.045) | 0.274 |
| 12-month | 1.024 (0.978 – 1.073) | 0.311 | -0.036 (-0.150 – 0.079) | 0.544 |
| **PM_2.5_** |  |  |  |  |
| 1-month | 0.998 (0.935 – 1.066) | 0.961 | -0.110 (-0.269 – 0.050) | 0.178 |
| 3-month | 1.046 (0.974 – 1.124) | 0.218 | -0.117 (-0.293 – 0.059) | 0.191 |
| 6-month | 1.081 (0.999 – 1.169) | 0.053 | -0.129 (-0.326 – 0.067) | 0.196 |
| 12-month | 1.045 (0.954 – 1.145) | 0.345 | -0.082 (-0.306 – 0.143) | 0.477 |
| **NO_2_** |  |  |  |  |
| 1-month | 1.000 (0.989 – 1.011) | 0.969 | -0.027 (-0.055 – 0.001) | 0.059 |
| 3-month | 1.002 (0.990 – 1.014) | 0.733 | -0.022 (-0.052 – 0.008) | 0.151 |
| 6-month | 1.004 (0.991 – 1.016) | 0.571 | -0.016 (-0.048 – 0.016) | 0.331 |
| 12-month | 1.000 (0.986 – 1.014) | 0.967 | -0.012 (-0.047 – 0.023) | 0.503 |
| **NO** |  |  |  |  |
| 1-month | 1.003 (0.990 – 1.017) | 0.651 | -0.010 (-0.041 – 0.021) | 0.522 |
| 3-month | 0.999 (0.986 – 1.012) | 0.863 | -0.008 (-0.041 – 0.025) | 0.632 |
| 6-month | 0.998 (0.985 – 1.010) | 0.714 | -0.004 (-0.036 – 0.029) | 0.832 |
| 12-month | 0.998 (0.985 – 1.011) | 0.752 | -0.008 (-0.044 – 0.027) | 0.637 |
| **NOx** |  |  |  |  |
| 1-month | 1.001 (0.994 – 1.007) | 0.868 | -0.015 (-0.030 – 0.000) | 0.054 |
| 3-month | 1.001 (0.994 – 1.008) | 0.732 | -0.014 (-0.031 – 0.004) | 0.125 |
| 6-month | 1.001 (0.994 – 1.009) | 0.682 | -0.011 (-0.029 – 0.007) | 0.251 |
| 12-month | 1.000 (0.993 – 1.007) | 0.991 | -0.008 (-0.027 – 0.011) | 0.395 |
| **SO_2_** |  |  |  |  |
| 1-month | 1.038 (0.980 – 1.098) | 0.206 | -0.058 (-0.201 – 0.084) | 0.424 |
| 3-month | **1.061 (1.000 – 1.125)*** | **0.048** | -0.057 (-0.206 – 0.093) | 0.456 |
| 6-month | **1.064 (1.002 – 1.130)*** | **0.042** | -0.081 (-0.235 – 0.072) | 0.300 |
| 12-month | 1.040 (0.979 – 1.104) | 0.202 | -0.065 (-0.216 – 0.087) | 0.401 |
| **CO** |  |  |  |  |
| 1-month | 1.059 (0.769 – 1.460) | 0.724 | **-0.904 (-1.733 – -0.075)*** | **0.033** |
| 3-month | 1.093 (0.770 – 1.551) | 0.618 | -0.854 (-1.750 – 0.042) | 0.062 |
| 6-month | 1.088 (0.755 – 1.567) | 0.652 | -0.685 (-1.637 – 0.267) | 0.159 |
| 12-month | 1.039 (0.703 – 1.537) | 0.847 | -0.706 (-1.732 – 0.319) | 0.177 |
| **O_3_** |  |  |  |  |
| 1-month | 0.998 (0.988 – 1.008) | 0.668 | -0.002 (-0.027 – 0.023) | 0.850 |
| 3-month | 1.007 (0.994 – 1.021) | 0.285 | 0.007 (-0.027 – 0.041) | 0.701 |
| 6-month | 1.008 (0.990 – 1.026) | 0.386 | 0.008 (-0.039 – 0.054) | 0.742 |
| 12-month | 1.008 (0.988 – 1.029) | 0.437 | 0.025 (-0.026 – 0.077) | 0.339 |
| **MCH** | | | | |
| **PM_10_** |  |  |  |  |
| 1-month | 1.003 (0.969 – 1.038) | 0.853 | -0.022 (-0.052 – 0.007) | 0.135 |
| 3-month | 1.023 (0.984 – 1.063) | 0.247 | -0.018 (-0.051 – 0.014) | 0.271 |
| 6-month | 1.032 (0.989 – 1.076) | 0.147 | -0.014 (-0.051 – 0.023) | 0.456 |
| 12-month | 1.014 (0.968 – 1.063) | 0.550 | -0.013 (-0.055 – 0.028) | 0.524 |
| **PM_2.5_** |  |  |  |  |
| 1-month | 0.982 (0.920 – 1.048) | 0.578 | -0.019 (-0.075 – 0.038) | 0.520 |
| 3-month | 1.019 (0.948 – 1.097) | 0.604 | -0.026 (-0.089 – 0.038) | 0.427 |
| 6-month | 1.047 (0.965 – 1.136) | 0.272 | -0.015 (-0.087 – 0.057) | 0.685 |
| 12-month | 1.007 (0.915 – 1.107) | 0.888 | -0.010 (-0.093 – 0.074) | 0.818 |
| **NO_2_** |  |  |  |  |
| 1-month | 1.006 (0.995 – 1.018) | 0.262 | **-0.012 (-0.022 – -0.003)*** | **0.013** |
| 3-month | 1.009 (0.997 – 1.021) | 0.128 | **-0.012 (-0.022 – -0.001)*** | **0.031** |
| 6-month | 1.012 (0.999 – 1.025) | 0.061 | **-0.011 (-0.023 – 0.000)*** | **0.048** |
| 12-month | 1.011 (0.997 – 1.025) | 0.110 | **-0.013 (-0.025 – 0.000)*** | **0.042** |
| **NO** |  |  |  |  |
| 1-month | 1.009 (0.996 – 1.023) | 0.174 | -0.005 (-0.016 – 0.006) | 0.386 |
| 3-month | 1.008 (0.995 – 1.022) | 0.218 | -0.006 (-0.018 – 0.005) | 0.276 |
| 6-month | 1.007 (0.995 – 1.020) | 0.259 | -0.006 (-0.017 – 0.005) | 0.296 |
| 12-month | 1.008 (0.994 – 1.021) | 0.252 | -0.008 (-0.021 – 0.004) | 0.169 |
| **NOx** |  |  |  |  |
| 1-month | 1.005 (0.998 – 1.011) | 0.158 | **-0.007 (-0.013 – -0.002)*** | **0.011** |
| 3-month | 1.007 (1.000 – 1.013) | 0.056 | **-0.008 (-0.014 – -0.002)*** | **0.012** |
| 6-month | 1.007 (1.000 – 1.014) | 0.053 | **-0.007 (-0.013 – -0.001)*** | **0.027** |
| 12-month | 1.006 (0.999 – 1.014) | 0.100 | **-0.007 (-0.014 – 0.000)*** | **0.038** |
| **SO_2_** |  |  |  |  |
| 1-month | 1.034 (0.975 – 1.096) | 0.263 | -0.041 (-0.091 – 0.010) | 0.116 |
| 3-month | **1.063 (1.001 – 1.130)*** | **0.047** | -0.051 (-0.104 – 0.003) | 0.062 |
| 6-month | **1.067 (1.004 – 1.135)*** | **0.037** | **-0.056 (-0.110 – -0.002)*** | **0.042** |
| 12-month | 1.050 (0.989 – 1.115) | 0.113 | -0.052 (-0.106 – 0.002) | 0.057 |
| **CO** |  |  |  |  |
| 1-month | 1.308 (0.942 – 1.817) | 0.109 | **-0.427 (-0.722 – -0.133)*** | **0.004** |
| 3-month | 1.339 (0.939 – 1.908) | 0.106 | **-0.362 (-0.679 – -0.045)*** | **0.025** |
| 6-month | 1.400 (0.963 – 2.035) | 0.078 | **-0.346 (-0.686 – -0.007)*** | **0.046** |
| 12-month | 1.383 (0.923 – 2.073) | 0.116 | **-0.406 (-0.774 – -0.039)*** | **0.030** |
| **O_3_** |  |  |  |  |
| 1-month | 0.992 (0.982 – 1.002) | 0.130 | 0.001 (-0.008 – 0.010) | 0.760 |
| 3-month | 0.996 (0.983 – 1.010) | 0.579 | 0.008 (-0.004 – 0.020) | 0.198 |
| 6-month | 0.997 (0.978 – 1.016) | 0.742 | 0.012 (-0.005 – 0.029) | 0.165 |
| 12-month | 0.994 (0.974 – 1.015) | 0.595 | **0.020 (0.002 – 0.039)*** | **0.033** |
| **MCHC** | | | | |
| **PM_10_** |  |  |  |  |
| 1-month | **0.964 (0.929 – 1.000)*** | **0.047** | -0.007 (-0.025 – 0.010) | 0.413 |
| 3-month | 0.975 (0.937 – 1.015) | 0.220 | -0.008 (-0.028 – 0.011) | 0.401 |
| 6-month | 0.987 (0.945 – 1.030) | 0.541 | -0.006 (-0.027 – 0.015) | 0.568 |
| 12-month | 0.985 (0.939 – 1.033) | 0.529 | -0.005 (-0.028 – 0.018) | 0.663 |
| **PM_2.5_** |  |  |  |  |
| 1-month | **0.927 (0.865 – 0.993)*** | **0.031** | -0.005 (-0.038 – 0.029) | 0.787 |
| 3-month | 0.944 (0.874 – 1.019) | 0.142 | -0.011 (-0.049 – 0.026) | 0.548 |
| 6-month | 0.962 (0.884 – 1.047) | 0.369 | -0.002 (-0.043 – 0.039) | 0.916 |
| 12-month | 0.964 (0.874 – 1.062) | 0.453 | 0.001 (-0.043 – 0.045) | 0.977 |
| **NO_2_** |  |  |  |  |
| 1-month | 0.999 (0.987 – 1.010) | 0.822 | -0.004 (-0.009 – 0.002) | 0.187 |
| 3-month | 1.003 (0.991 – 1.015) | 0.663 | -0.003 (-0.009 – 0.003) | 0.279 |
| 6-month | 1.007 (0.995 – 1.020) | 0.251 | -0.004 (-0.010 – 0.003) | 0.284 |
| 12-month | 1.010 (0.996 – 1.024) | 0.150 | -0.005 (-0.012 – 0.002) | 0.183 |
| **NO** |  |  |  |  |
| 1-month | 1.007 (0.993 – 1.021) | 0.349 | -0.002 (-0.008 – 0.005) | 0.642 |
| 3-month | 1.008 (0.993 – 1.023) | 0.291 | -0.001 (-0.008 – 0.006) | 0.829 |
| 6-month | 1.008 (0.994 – 1.023) | 0.253 | -0.001 (-0.008 – 0.006) | 0.685 |
| 12-month | 1.009 (0.995 – 1.024) | 0.217 | -0.004 (-0.011 – 0.004) | 0.333 |
| **NOx** |  |  |  |  |
| 1-month | 1.001 (0.995 – 1.008) | 0.688 | -0.002 (-0.006 – 0.001) | 0.137 |
| 3-month | 1.003 (0.996 – 1.010) | 0.362 | -0.002 (-0.006 – 0.001) | 0.202 |
| 6-month | 1.005 (0.998 – 1.012) | 0.183 | -0.003 (-0.006 – 0.001) | 0.175 |
| 12-month | 1.006 (0.998 – 1.013) | 0.124 | -0.003 (-0.007 – 0.001) | 0.120 |
| **SO_2_** |  |  |  |  |
| 1-month | 1.018 (0.959 – 1.080) | 0.560 | -0.016 (-0.045 – 0.013) | 0.274 |
| 3-month | 1.048 (0.985 – 1.114) | 0.140 | -0.020 (-0.051 – 0.010) | 0.194 |
| 6-month | 1.040 (0.978 – 1.107) | 0.212 | -0.019 (-0.050 – 0.012) | 0.226 |
| 12-month | 1.049 (0.986 – 1.116) | 0.131 | -0.019 (-0.049 – 0.012) | 0.224 |
| **CO** |  |  |  |  |
| 1-month | 1.088 (0.782 – 1.514) | 0.615 | -0.074 (-0.242 – 0.094) | 0.385 |
| 3-month | 1.160 (0.810 – 1.661) | 0.418 | -0.055 (-0.239 – 0.129) | 0.560 |
| 6-month | 1.288 (0.887 – 1.870) | 0.183 | -0.044 (-0.239 – 0.152) | 0.663 |
| 12-month | 1.346 (0.903 – 2.006) | 0.145 | -0.061 (-0.272 – 0.150) | 0.572 |
| **O_3_** |  |  |  |  |
| 1-month | **0.989 (0.979 – 0.999)*** | **0.035** | 0.002 (-0.003 – 0.007) | 0.389 |
| 3-month | 0.988 (0.974 – 1.001) | 0.077 | -0.001 (-0.008 – 0.006) | 0.804 |
| 6-month | 0.989 (0.972 – 1.007) | 0.250 | 0.002 (-0.007 – 0.012) | 0.594 |
| 12-month | 0.988 (0.969 – 1.008) | 0.252 | 0.008 (-0.003 – 0.018) | 0.144 |

COPD, chronic obstructive pulmonary disease; RBC, red blood cell; HGB, hemoglobin; MCV, mean corpuscular volume; MCH, mean corpuscular hemoglobin; MCHC, mean corpuscular hemoglobin concentration; PM_10_, particulate matter with an aerodynamic diameter ≤ 10 µm; PM_2.5_, particulate matter with an aerodynamic diameter ≤ 2.5 µm; NO_2_, nitrogen dioxide; NO, nitric oxide (nitrogen monoxide); NOx, nitrogen oxides; SO_2_, sulfur dioxide; CO, carbon monoxide; O_3_, ozone. Odds ratios (ORs) indicate the risk based on logistic regression models, per 10 µg/m^3^ increase in PM_10_ and PM_2.5_ concentrations, and per 1-unit increase in concentrations of other pollutants

^†^adjusted for age, sex, body mass index (BMI), smoking experience, and principal components (PC1-PC10)

**p* <0.05

**Figure S1. Manhattan and QQ plots of COPD. λgc = 1.046**

**Table S14. GWAS lead loci for COPD**

| **Lead SNP** | **CHR** | **BP** | **A1** | **OR** | **p-value** | **Clump size** | **Gene** | **Functional annotation** | **Previous GWAS traits** | **Known/novel** |
| --- | --- | --- | --- | --- | --- | --- | --- | --- | --- | --- |
| rs4808295 | 19 | 21747515 | A | 0.8646 | 1.63e-10 | 151 | ZNF100 (mapped) | Intron variant | No direct match in GWAS Catalog | No direct prior association identified for this SNP in GWAS Catalog |
| rs138998202 | 1 | 18387505 | T | 1.434 | 1.91e-08 | 14 | LOC124903865 (mapped) | Intron variant | No direct match in GWAS Catalog | No direct prior association identified for this SNP in GWAS Catalog |

**Figure S2. Manhattan and QQ plots of COPD with QC. Λgc = 1.046**

**Table S15. Main versus sensitivity (QC) comparison for LD clumping of COPD**

| **Locus** | **Lead SNP main** | **Clump size main** | **Lead SNP QC** | **Clump size QC** | **OR** | **p-value** | **Interpretation** |
| --- | --- | --- | --- | --- | --- | --- | --- |
| **Genome-wide clumping** | | | | | | | |
| chr19:21747515 | rs4808295 | 151 | rs4808295 | 116 | 0.8646 | 1.63e-10 | Stable |
| chr1:18387505 | rs138998202 | 14 | NA | NA | 1.434 | 1.91e-08 | Not replicated |
| **Exploratory clumping** | | | | | | | |
| chr10:128931599 | rs74700044 | 57 | NA | NA | 4.071 | 6.37e-08 | Not replicated |
| chr2:9222068 | rs144605342 | 4 | NA | NA | 0.6444 | 2.50e-07 | Not replicated |
| chr15:42621327 | rs755727176 | 11 | NA | NA | 1.959 | 2.69e-07 | Not replicated |
| chr11:105458082 | rs117797321 | 8 | NA | NA | 0.652 | 3.21e-07 | Not replicated |
| chr7:32288133 | rs188677997 | 0 | NA | NA | 5.917 | 5.14e-07 | Not replicated |
| chr15:41699939 | rs73407225 | 7 | NA | NA | 1.796 | 5.18e-07 | Not replicated |
| chr5:46094830 | rs558209432 | 3 | NA | NA | 2.14 | 6.50e-07 | Not replicated |
| chr15:42961683 | rs952375350 | 4 | NA | NA | 2.037 | 6.52e-07 | Not replicated |
| chr3:4843555 | rs146067816 | 3 | NA | NA | 0.517 | 7.92e-07 | Not replicated |
| chr12:114857306 | rs188458071 | 4 | NA | NA | 0.693 | 9.67e-07 | Not replicated |
| chr15:43769048 | rs919426315 | 3 | NA | NA | 2.007 | 1.03e-06 | Not replicated |
| chr7:64297934 | rs1480736112 | 1 | NA | NA | 4.6 | 1.06e-06 | Not replicated |
| chr9:33063784 | rs190632637 | 1 | NA | NA | 2.803 | 1.30e-06 | Not replicated |
| chr6:155450008 | rs12196156 | 11 | NA | NA | 1.242 | 1.46e-06 | Not replicated |
| chr1:36590319 | rs78811708 | 4 | NA | NA | 0.7239 | 1.65e-06 | Not replicated |
| chr13:99528503 | rs146818182 | 6 | NA | NA | 1.738 | 1.65e-06 | Not replicated |
| chr10:45618046 | rs1265995675 | 6 | NA | NA | 3.586 | 1.67e-06 | Not replicated |
| chr4:136186299 | rs145118002 | 91 | NA | NA | 1.298 | 1.70e-06 | Not replicated |
| chr6:80267499 | rs1195691320 | 3 | NA | NA | 3.712 | 1.76e-06 | Not replicated |
| chr15:42285939 | rs886986442 | 5 | NA | NA | 2.012 | 1.84e-06 | Not replicated |
| chr15:98817023 | rs556048468 | 0 | NA | NA | 3.306 | 1.88e-06 | Not replicated |
| chr10:129977988 | rs1331694827 | 5 | NA | NA | 2.533 | 2.08e-06 | Not replicated |
| chr15:43311135 | rs1049446867 | 3 | NA | NA | 1.959 | 2.13e-06 | Not replicated |
| chr3:162711435 | rs1316407187 | 0 | NA | NA | 4.454 | 2.16e-06 | Not replicated |
| chr17:7689055 | rs34067256 | 19 | NA | NA | 0.7951 | 2.32e-06 | Not replicated |
| chr5:138625447 | rs141113461 | 3 | NA | NA | 0.4719 | 2.46e-06 | Not replicated |
| chr1:38790729 | rs59422224 | 9 | NA | NA | 1.177 | 2.59e-06 | Not replicated |
| chr3:107809030 | rs570955230 | 5 | NA | NA | 2.125 | 2.84e-06 | Not replicated |
| chr12:9966400 | rs2594083 | 30 | NA | NA | 0.7821 | 3.00e-06 | Not replicated |
| chr11:86253169 | rs1467355546 | 1 | NA | NA | 2.659 | 3.02e-06 | Not replicated |
| chr5:53844625 | rs1338884053 | 2 | NA | NA | 2.894 | 3.35e-06 | Not replicated |
| chr1:112899549 | rs1264347184 | 0 | NA | NA | 2.305 | 3.52e-06 | Not replicated |
| chr15:42033899 | rs535293895 | 0 | NA | NA | 1.878 | 3.77e-06 | Not replicated |
| chr4:65294457 | rs12501311 | 93 | rs12501311 | 89 | 1.091 | 3.86e-06 | Stable |
| chr12:79958146 | rs778984958 | 3 | NA | NA | 2.568 | 3.90e-06 | Not replicated |
| chr4:58034223 | rs537202772 | 7 | NA | NA | 2.016 | 3.98e-06 | Not replicated |
| chr12:62695779 | rs558559508 | 7 | NA | NA | 2.761 | 4.68e-06 | Not replicated |
| chr1:28702162 | rs1486133956 | 6 | NA | NA | 2.68 | 4.73e-06 | Not replicated |
| chr11:27279204 | rs555320973 | 9 | NA | NA | 2.143 | 4.73e-06 | Not replicated |
| chr13:39089188 | rs1393566059 | 8 | NA | NA | 3.962 | 4.74e-06 | Not replicated |
| chr4:100556078 | rs370585058 | 1 | NA | NA | 2.8 | 5.17e-06 | Not replicated |
| chr7:20690628 | rs75204054 | 0 | rs75204054 | 0 | 0.8967 | 5.20e-06 | Stable |
| chr1:28449021 | rs371252350 | 0 | NA | NA | 2.795 | 5.34e-06 | Not replicated |
| chr15:44072218 | rs1029028673 | 3 | NA | NA | 1.962 | 5.53e-06 | Not replicated |
| chr3:89012634 | rs138310759 | 2 | NA | NA | 2.071 | 5.63e-06 | Not replicated |
| chr11:74723872 | rs558454061 | 5 | NA | NA | 3.196 | 6.19e-06 | Not replicated |
| chr1:175502337 | rs145963709 | 5 | NA | NA | 0.6752 | 6.64e-06 | Not replicated |
| chr6:82885057 | rs79873708 | 3 | NA | NA | 0.4148 | 6.74e-06 | Not replicated |
| chr2:195426632 | rs1400906335 | 8 | NA | NA | 2.192 | 6.87e-06 | Not replicated |
| chr20:48841805 | rs151237928 | 5 | NA | NA | 2.188 | 7.23e-06 | Not replicated |
| chr6:158495007 | rs145122588 | 20 | NA | NA | 1.84 | 7.30e-06 | Not replicated |
| chr20:59656820 | rs570720688 | 1 | NA | NA | 2.247 | 7.53e-06 | Not replicated |
| chr3:11045714 | rs148111814 | 4 | NA | NA | 1.918 | 7.60e-06 | Not replicated |
| chr10:26190836 | rs188946897 | 2 | NA | NA | 2.532 | 7.78e-06 | Not replicated |
| chr18:49344470 | rs143745276 | 8 | NA | NA | 2.453 | 8.74e-06 | Not replicated |
| chr3:120758892 | rs143659182 | 7 | NA | NA | 1.89 | 8.81e-06 | Not replicated |
| chr14:80643045 | rs188237915 | 68 | NA | NA | 2.393 | 8.81e-06 | Not replicated |
| chr2:163561687 | rs1237735750 | 1 | NA | NA | 3.595 | 8.88e-06 | Not replicated |
| chr12:62237830 | rs571865884 | 4 | NA | NA | 2.41 | 8.98e-06 | Not replicated |
| chr9:71372980 | rs149923165 | 3 | NA | NA | 0.5626 | 9.38e-06 | Not replicated |
| chr15:83390201 | rs943189799 | 4 | NA | NA | 2.645 | 9.45e-06 | Not replicated |
| chr8:137606508 | rs549027231 | 1 | NA | NA | 4.546 | 9.82e-06 | Not replicated |
| chr10:98331042 | rs148419322 | 8 | NA | NA | 1.645 | 9.82e-06 | Not replicated |
| chr18:60680828 | rs550490354 | 27 | NA | NA | 1.709 | 9.87e-06 | Not replicated |
| chr9:80280116 | rs140135608 | 9 | NA | NA | 0.4215 | 9.99e-06 | Not replicated |

**Figure S3. Manhattan and QQ plots of anemia in COPD. λgc = 0.897**

**Table S16. GWAS lead loci for anemia in COPD**

| **Lead SNP** | **CHR** | **BP** | **A1** | **OR** | **p-value** | **Clump size** | **Gene** | **Functional annotation** | **Previous GWAS traits** | **Known/novel** |
| --- | --- | --- | --- | --- | --- | --- | --- | --- | --- | --- |
| rs375498857 | 16 | 377772 | A | 5.550 | 9.16e-29 | 1 | PGAP6 (mapped) | Missense / 5′UTR variant | Hemoglobin, erythrocyte count, hematocrit, anemia | Previously reported for related hematologic traits |
| rs183154008 | 11 | 5089524 | C | 9.462 | 2.4e-23 | 6 | OR52E1 (nearest) | Intergenic variant | No direct match in GWAS Catalog | No direct prior association identified for this SNP in GWAS Catalog |
| rs11038031 | 11 | 5542318 | G | 6.054 | 3.36e-12 | 7 | OR52H1, OR52V1P (mapped) | Intron variant | Hemoglobin, hematocrit, erythrocyte count | Previously reported for related hematologic traits |

**Figure S4. GWAS and QQ plot of anemia in COPD with QC. λgc = 0.986**

**Table S17. Main versus sensitivity (QC) comparison for LD clumping of anemia in COPD**

| **Locus** | **Lead SNP main** | **Clump size main** | **Lead SNP QC** | **Clump size QC** | **OR** | **p-value** | **Interpretation** |
| --- | --- | --- | --- | --- | --- | --- | --- |
| **Genome-wide clumping** | | | | | | | |
| chr16:377772 | rs375498857 | 1 | NA | NA | 5.55 | 9.16e-29 | Not replicated |
| chr11:5089524 | rs183154008 | 6 | NA | NA | 9.462 | 2.40e-23 | Not replicated |
| chr11:5542318 | rs11038031 | 7 | NA | NA | 6.054 | 3.36e-12 | Not replicated |
| **Exploratory clumping** | | | | | | | |
| chr11:4517579 | rs1283995601 | 6 | NA | NA | 9.582 | 5.41e-08 | Not replicated |
| chr1:240538746 | rs368716054 | 1 | NA | NA | 16.05 | 1.32e-07 | Not replicated |
| chr17:50421058 | rs149429335 | 6 | NA | NA | 5.443 | 1.42e-07 | Not replicated |
| chr11:4568936 | rs753059881 | 4 | NA | NA | 8.239 | 1.53e-07 | Not replicated |
| chr3:104439520 | rs559982306 | 3 | NA | NA | 11.87 | 1.86e-07 | Not replicated |
| chr2:69643695 | rs1225308848 | 2 | NA | NA | 9.716 | 2.15e-07 | Not replicated |
| chr19:19232634 | rs139023873 | 5 | NA | NA | 5.339 | 2.75e-07 | Not replicated |
| chr1:53597068 | rs138560471 | 26 | NA | NA | 1.992 | 5.27e-07 | Not replicated |
| chr15:27609604 | rs1257362608 | 1 | NA | NA | 7.161 | 9.58e-07 | Not replicated |
| chr17:44227994 | rs1173401294 | 3 | NA | NA | 11.09 | 1.13e-06 | Not replicated |
| chr10:120917831 | rs76518733 | 30 | NA | NA | 1.964 | 1.38e-06 | Not replicated |
| chr16:48182812 | rs532650705 | 5 | NA | NA | 9.241 | 1.43e-06 | Not replicated |
| chr1:183029751 | rs143860562 | 4 | NA | NA | 3.203 | 1.46e-06 | Not replicated |
| chr11:125333509 | rs575737933 | 4 | NA | NA | 4.906 | 1.75e-06 | Not replicated |
| chr11:71429876 | rs1425672270 | 0 | NA | NA | 11.51 | 1.97e-06 | Not replicated |
| chr13:31434995 | rs377722228 | 3 | NA | NA | 5.635 | 2.02e-06 | Not replicated |
| chr18:78399031 | rs1211002364 | 5 | NA | NA | 10.62 | 2.21e-06 | Not replicated |
| chr7:147962150 | rs913482008 | 3 | NA | NA | 23.29 | 2.52e-06 | Not replicated |
| chr11:4108436 | rs372137391 | 2 | NA | NA | 5.176 | 2.54e-06 | Not replicated |
| chr1:95827055 | rs879608133 | 3 | NA | NA | 13.8 | 2.61e-06 | Not replicated |
| chr6:122909968 | rs889516527 | 10 | NA | NA | 11.36 | 2.91e-06 | Not replicated |
| chr13:84508832 | rs576750413 | 1 | NA | NA | 9.113 | 2.95e-06 | Not replicated |
| chr13:63844053 | rs181653936 | 5 | NA | NA | 3.486 | 3.33e-06 | Not replicated |
| chr6:25621788 | rs540860373 | 2 | NA | NA | 34.37 | 3.55e-06 | Not replicated |
| chr1:183815735 | rs1306707067 | 7 | NA | NA | 5.267 | 4.25e-06 | Not replicated |
| chr2:8119176 | rs146049836 | 0 | NA | NA | 9.572 | 4.40e-06 | Not replicated |
| chr17:10707686 | rs570466403 | 9 | NA | NA | 3.308 | 4.47e-06 | Not replicated |
| chr2:144949510 | rs563947493 | 1 | NA | NA | 7.466 | 4.48e-06 | Not replicated |
| chr11:9559032 | rs570777575 | 5 | NA | NA | 13.99 | 4.50e-06 | Not replicated |
| chr9:129786046 | rs1003738410 | 3 | NA | NA | 6.597 | 4.63e-06 | Not replicated |
| chr21:23563494 | rs1266336153 | 3 | NA | NA | 11.66 | 4.99e-06 | Not replicated |
| chr4:45872164 | rs1311267702 | 9 | NA | NA | 5.874 | 5.00e-06 | Not replicated |
| chr12:75354111 | rs1026414732 | 4 | NA | NA | 4.548 | 5.05e-06 | Not replicated |
| chr11:9886696 | rs556358490 | 1 | NA | NA | 12.67 | 5.14e-06 | Not replicated |
| chr2:148212495 | rs10497032 | 292 | rs10497032 | 236 | 1.322 | 5.33e-06 | Stable |
| chr3:59306724 | rs1272228892 | 3 | NA | NA | 8.82 | 5.65e-06 | Not replicated |
| chr11:79491094 | rs143334817 | 8 | NA | NA | 3.594 | 5.96e-06 | Not replicated |
| chr17:53750448 | rs541890512 | 5 | NA | NA | 5.998 | 5.97e-06 | Not replicated |
| chr2:145614348 | rs187708338 | 5 | NA | NA | 8.537 | 6.04e-06 | Not replicated |
| chr4:118972827 | rs199530237 | 0 | NA | NA | 12.41 | 6.08e-06 | Not replicated |
| chr2:22227698 | chr2:22227698_G_T | 1 | NA | NA | 10.75 | 6.21e-06 | Not replicated |
| chr2:158980905 | rs186153295 | 3 | NA | NA | 5.785 | 6.34e-06 | Not replicated |
| chr8:65460772 | rs534012672 | 0 | NA | NA | 5.861 | 6.38e-06 | Not replicated |
| chr17:44579393 | rs9910962 | 39 | NA | NA | 1.638 | 6.68e-06 | Not replicated |
| chr5:151204656 | rs561579578 | 6 | NA | NA | 5.396 | 6.73e-06 | Not replicated |
| chr2:146515522 | rs187458914 | 3 | NA | NA | 8.244 | 7.16e-06 | Not replicated |
| chr3:31823352 | rs554945420 | 3 | NA | NA | 13.7 | 7.58e-06 | Not replicated |
| chr10:106310090 | rs140136508 | 6 | NA | NA | 4.95 | 7.62e-06 | Not replicated |
| chr2:61142313 | rs568354103 | 7 | NA | NA | 4.217 | 7.64e-06 | Not replicated |
| chr4:161090005 | rs187350757 | 28 | NA | NA | 4.815 | 7.70e-06 | Not replicated |
| chr5:154986396 | rs59025588 | 299 | NA | NA | 1.714 | 7.91e-06 | Not replicated |
| chr15:33073689 | rs537908685 | 6 | NA | NA | 4.886 | 8.15e-06 | Not replicated |
| chr6:18812083 | rs1221695841 | 1 | NA | NA | 6.729 | 8.39e-06 | Not replicated |
| chr6:109962067 | rs146682592 | 4 | NA | NA | 2.72 | 8.40e-06 | Not replicated |
| chr7:147163129 | rs569097758 | 2 | NA | NA | 13.29 | 8.40e-06 | Not replicated |
| chr4:11813142 | rs570068880 | 5 | NA | NA | 4.498 | 9.02e-06 | Not replicated |
| chr7:101860711 | rs1055875924 | 1 | NA | NA | 10.13 | 9.04e-06 | Not replicated |
| chr11:66435737 | rs149871130 | 8 | NA | NA | 4.05 | 9.74e-06 | Not replicated |

**References**

[1]	Global Initiative for Chronic Obstructive Lung Disease. Global strategy for the diagnosis, management, and prevention of chronic obstructive pulmonary disease. 2026.

[2]	Choi SW, Mak TS, O'Reilly PF. Tutorial: a guide to performing polygenic risk score analyses. Nat Protoc. 2020;15(9):2759-2772.

[3]	Shrine N, Guyatt AL, Erzurumluoglu AM, et al. New genetic signals for lung function highlight pathways and chronic obstructive pulmonary disease associations across multiple ancestries. Nature Genetics. 2019;51(3):481-493.

[4]	Tanigawa Y, Qian J, Venkataraman G, et al. Significant sparse polygenic risk scores across 813 traits in UK Biobank. PLOS Genetics. 2022;18(3):e1010105.

[5]	Moreno-Grau S, Vernekar M, Lopez-Pineda A, et al. Polygenic risk score portability for common diseases across genetically diverse populations. Human Genomics. 2024;18(1):93.

[6]	Li S, Gui J, Passarelli MN, et al. Genome-Wide and Transcriptome-Wide Association Studies on Northern New England and Ohio Amyotrophic Lateral Sclerosis Cohorts. Neurol Genet. 2024;10(5):e200188.
